# Functional Analysis of RNA Interference-Related Soybean Pod Borer (*Lepidoptera*) Genes Based on Transcriptome Sequences

**DOI:** 10.3389/fphys.2018.00383

**Published:** 2018-05-03

**Authors:** Fanli Meng, Mingyu Yang, Yang Li, Tianyu Li, Xinxin Liu, Guoyue Wang, Zhanchun Wang, Xianhao Jin, Wenbin Li

**Affiliations:** Key Laboratory of Soybean Biology in Chinese Ministry of Education, Northeast Agricultural University, Harbin, China

**Keywords:** soybean pod borer, RNA interference, RNAi-of-RNAi assay, systemic RNA interference deficient (SID), scavenger receptor-mediated endocytosis

## Abstract

RNA interference (RNAi) is useful for controlling pests of agriculturally important crops. The soybean pod borer (SPB) is the most important soybean pest in Northeastern Asia. In an earlier study, we confirmed that the SPB could be controlled via transgenic plant-mediated RNAi. Here, the SPB transcriptome was sequenced to identify RNAi-related genes, and also to establish an RNAi-of-RNAi assay system for evaluating genes involved in the SPB systemic RNAi response. The core RNAi genes, as well as genes potentially involved in double-stranded RNA (dsRNA) uptake were identified based on SPB transcriptome sequences. A phylogenetic analysis and the characterization of these core components as well as dsRNA uptake related genes revealed that they contain conserved domains essential for the RNAi pathway. The results of the RNAi-of-RNAi assay involving *Laccas*e 2 (a critical cuticle pigmentation gene) as a marker showed that genes encoding the sid-like (*Sil1*), scavenger receptor class C (*Src*), and scavenger receptor class B (*Srb3* and *Srb4*) proteins of the endocytic pathway were required for SPB cellular uptake of dsRNA. The SPB response was inferred to contain three functional small RNA pathways (i.e., miRNA, siRNA, and piRNA pathways). Additionally, the SPB systemic RNA response may rely on systemic RNA interference deficient transmembrane channel-mediated and receptor-mediated endocytic pathways. The results presented herein may be useful for developing RNAi-mediated methods to control SPB infestations in soybean.

## Introduction

RNA interference (RNAi) is an evolutionarily conserved mechanism that refers to a set of molecular processes in which small RNA (sRNA) molecules exert a silencing effect on complementary mRNAs (Miyata et al., 2014; Ganbaatar et al., 2017). The RNAi-based technology has recently been exploited to control insect pests of agriculturally important crops (Mao and Zeng, 2014). Previous studies revealed that RNA-mediated gene silencing may involves the piwi-interacting RNA (piRNA), microRNA (miRNA), or small interfering RNA (siRNA) pathways (Tomoyasu et al., 2008; Dowling et al., 2017). The application of RNAi technology to control pests is based on the introduction of exogenous double-stranded RNA (dsRNA) into insects to silence the target genes, thereby activating the siRNA pathway (Joga et al., 2016). The exogenous long dsRNA molecules are introduced into insect cells and are cleaved into siRNA consisting of 21–23 base pairs (bp) by Dicer-2 RNase III (Zhao et al., 2015). The dsRNA-binding motif protein, R2D2, ensures siRNA is properly loaded into the RNA-induced silencing complex (RISC) (Nishida et al., 2013; Swevers et al., 2013). The RISC-RNA complex combines with Argonaute2 to form the RISC functional center (Dueck and Meister, 2014). Using siRNA as a guide, the RISC locates the target mRNA, which is then cleaved by Argonaute2 to ultimately downregulate protein expression (Azlan et al., 2016).

To date, the application of RNAi technology to control pests has been limited partly because of RNAi differences among diverse insects. Some insects, including several pests, exhibit a robust systemic RNAi response (Mao et al., 2011; Powell et al., 2017). RNA interference is induced by dsRNA molecules (i.e., feeding RNAi or plant-mediated RNAi), with long-term activities that are mediated by genes that may be passed to subsequent generations (Rangasamy and Siegfried, 2012). However, some insects exhibit relatively low or inefficient RNAi responses, resulting in temporary gene silencing (Shukla et al., 2016). Systemic RNAi events consist of two major steps. Extracellular dsRNA is taken up by cells from the extracellular environment and then processed by core RNAi machinery. Secondly, the silencing signal is transmitted from one cell to another or from one tissue to another (Joga et al., 2016). Although there are some variations, the core RNAi components, such as Argonaute2 and Dicer2, contain conserved domains essential for functions in insect RNAi pathways (Zhao et al., 2015). Therefore, diverse molecular mechanisms in various insects are responsible for the differences in the ability to elicit a robust systemic RNAi response. Several factors influence the differences in RNAi efficiencies (Taning et al., 2016). In addition to the presence of dsRNA-degrading enzymes (Christiaens et al., 2014), the uptake of dsRNA by insect cells is critical for robust systemic RNAi responses (Cappelle et al., 2016).

Two different dsRNA uptake pathways have been described in insect pests so far, namely the transmembrane Sid-1 channel protein-mediated and the endocytic pathways (Joga et al., 2016). Sid-1 is a multi-span transmembrane protein that related to the systemic spread of the RNAi response in the nematode *Caenorhabditis elegans* (Dowling et al., 2017). Although Sid-1 orthologs have been detected in most insect species, other transmembrane proteins (Sid-2, Sid-5, and the tyrosine kinase SID-3) have not been identified in insect species with sequenced genomes (Joga et al., 2016). *Drosophila* species lack a sid-1 homolog, but dsRNA uptake through the endocytic pathway has been confirmed in Drosophila S2 cells. Pattern recognition receptors (scavenger receptors) are important for the uptake of dsRNA by receptor-mediated endocytosis. When two scavenger receptors (SR-CI and Eater) are simultaneously silenced by RNAi in S2 cell, dsRNA uptake is downregulated by more than 90% (Ulvila et al., 2006). Scavenger receptors may function as the receptors of dsRNA molecules during the endocytic process (Miyata et al., 2014).

Soybean pod borer [SPB; *Leguminivora glycinivorella* (Matsumura) (Lepidoptera: Tortricidae)] is the most important soybean pest in Northeastern Asia, causing annual yield losses that are estimated to exceed $10 million. Insecticides have been used to control the SPB over the past three decades. However, SPB larvae are relatively well protected from insecticides under a closed canopy. Interestingly, the SPB exhibits a robust systemic RNAi response. The gene encoding SPB *Spbtry1* (serine protease) can be silenced via the feeding of *Spbtry1* dsRNA (Meng et al., 2017a). We previously observed that three transgenic soybean plants expressing the dsRNA of the SPB ribosomal protein P0 genes were more resistant to the SPB than wild-type plants. These results further confirmed that RNAi-based technology might be useful for controlling the SPB (Meng et al., 2017b). Nevertheless, the molecules and pathways involved in the SPB systemic RNAi processes have not been characterized.

An RNAi-of-RNAi assay system has been developed for screening genes involved in the RNAi pathways of the red flour beetle (*Tribolium castaneum*) (Tomoyasu et al., 2008), western corn rootworm (*Diabrotica virgifera virgifera*) (Miyata et al., 2014), and Colorado potato beetle (*Leptinotarsa decemlineata*) (Cappelle et al., 2016). This assay system consists of two RNAi feeding experiments. First, the dsRNA for a candidate gene involved in systemic RNAi is fed to larvae for 3 days, and the resulting larvae were then fed with dsRNA targeting a “reporter” gene. Changes of the silencing efficiency of the reporter gene indicated the involvement of the candidate gene in the systemic RNAi response (Miyata et al., 2014; Cappelle et al., 2016).

To comprehensively evaluate the genes associated with SPB systemic RNAi, the transcriptomes of SPB larvae fed with the pods of transgenic *SpbP0* dsRNA-expressing (dsSpbP0) or wild-type “Dongnong50” (DN50) soybean plants were compared. First, the SPB homologs of genes involved in RNA-mediated gene silencing and dsRNA uptake were identified. Second, an RNAi-of-RNAi assay system was established to evaluate the involvement of the Sid-1 channel protein-mediated and endocytic pathways in the SPB systemic RNAi responses. The aims were to identity the SPB systemic RNAi genes and to provide valuable information for the development of RNAi-mediated methods effective against the SPB.

## Materials and methods

### Insect rearing

The insects used in this study were from a continuous SPB colony that has been reared on an artificial diet since 2010 (Meng et al., 2017a). The T3 dsSpbP0 and wild-type DN50 plants were grown in a greenhouse at 24 ± 1°C with 60% relative humidity under a 16-h light/8-h dark cycle (Meng et al., 2017b). At the R5 soybean stage (fully developed pods), three replicates of 50 first-instar larvae were reared on soybean pods of T3 dsSpbP0 and DN50 plants (provided by the Key Laboratory of Soybean Biology of the Chinese Education Ministry, Harbin, China). The larvae fed on transgenic and wild-type plants were collected 3 days later. All samples were immediately frozen in liquid nitrogen and stored at −80°C until used.

Total RNA was extracted from pooled larvae using the TRIzol reagent (Invitrogen, Carlsbad, CA, USA). Residual genomic DNA was removed from the RNA extracts by adding 2 μl (1 mg/μl) DNAse I (Invitrogen) and incubating for 30 min at 37°C. The RNA was further purified using the RNeasy MinElute Clean-up Kit (Qiagen, Valencia, CA, USA). The cDNA library constructed as described by Chen et al. (2014) was sequenced using the Illumina HiSeq 2000 system.

### Sequence analysis and assembly

Clean data were obtained by removing reads containing adapter sequences, poor quality sequences (reads with ambiguous bases), and reads with more than 10% of the bases with a Q score below 20. The clean reads were assembled into unigenes using the Trinity method (http://trinityrnaseq.sourceforge.net/; Han et al., 2013). The unigenes from six samples were combined to create the SPB unigene database. All raw transcriptome data have been deposited in the NIH Short Read Archive accession numbers (SRR5985986, SRR5985987, SRR5985984, SRR5985985, SRR5985988, and SRR5985989).

### Functional annotations

Unigenes were aligned with sequences in the GenBank database as well as in the NR (http://www.ncbi.nlm.nih.gov/), Swiss-Prot (http://www.ebi.ac.uk/uniprot/), KEGG (http://www.genome.jp/kegg/), and COG (http://www.ncbi.nlm.nih.gov/COG/) databases using the BLAST online tool. The unigenes were functionally annotated based on GO terms (http://www.geneontology.org). All BLAST searches were completed with a cut-off e-value of 10^−5^ (Chen et al., 2014).

### Identification of RNA interference-related genes

A list of RNAi-related genes was chosen based on the available relevant literature. The genes were grouped into the following categories: RNAi core machinery, auxiliary factors, dsRNA uptake, antiviral RNAi, and nucleases (Table S5). *C. elegans, B. mori, T. castaneum*, and *D. melanogaster* homologs of these genes were identified in the GenBank database. The tBLASTn tool was used to complete sequence similarity searches of the SPB transcriptome database (Swevers et al., 2013; Taning et al., 2016).

### Phylogenetic analysis

Amino acid sequences were aligned with the Multiple Alignment program (http://www.ebi.ac.uk/clustalw/index.html) and phylogenetic analyses were constructed by MEGA 6.0 using neighbor joining method with 1,000 bootstrap replicates (Tamura et al., 2013).

### Domain analysis

The architecture of protein domains was analyzed with ScanProsite (http://prosite.expasy.org/scanprosite/), while the Sil proteins were analyzed using the TMHMM Server 2.0 (http://www.cbs.dtu.dk/services/TMHMM/).

### dsRNA synthesis

The dsRNA was synthesized using SPB larval cDNA, appropriate T7 primers (Table S6), and the T7 RiboMAX Express Large Scale RNA Production System (Promega, WI, USA). For the negative control, GFP dsRNA was prepared from the pCAMBIA1302 expression vector.

### dsRNA feeding

The first-instar larvae (1 day after hatching) were provided with dsRNA of target gene (Sil or scavenger receptor gene) in an artificial diet. The final concentration of dsRNA in the diet was 5 μg/ml. Control larvae were treated with the same concentration of GFP dsRNA. Each treatment and control were carried out on 50 larvae and replicated in triplicate. The larvae were reared at 26°C, humidity 80–90%, and 16:8 h L:D for 6 days, the expression levels of target genes were analyzed by qRT-PCR (Meng et al., 2017a).

To complete the RNAi-of-RNAi assays, a method for feeding larvae dsRNA was developed. At day 0, the dsRNA of the target gene was mixed with the artificial diet for a final dsRNA concentration of 5 μg/ml. The first-instar larvae (1 day after hatching) were fed the modified artificial diet. Three days later, the larvae were fed 5 μg dsRNA of the reporter gene. On day 6 or 7 (after the first larval molt), the larval body colors were recorded, after which the insects were frozen in liquid nitrogen and kept at −80°C.

Five different control groups were necessary to assess the RNAi response. One of the three negative controls consisted of larvae fed 5 μg GFP dsRNA on days 0 and 3, the second negative control comprised larvae fed 5 μg GFP dsRNA on day 0, but 5 μg reporter gene dsRNA on day 3, while the third negative control consisted of larvae fed 5 μg target gene dsRNA on day 0, but 5 μg GFP dsRNA on day 3. One of the two positive controls consisted of larvae fed 5 μg SPBAgro2 dsRNA or SPBDir2 dsRNA on day 0, but 5 μg reporter gene dsRNA on day 3. The pigmentation and mortality of the larvae fed only GFP, SPBAgro2, or SPBDir2 dsRNA were unaffected. Analyses were completed with three replicates of 50 larvae. The dsRNA artificial feed was provided fresh every day.

### Quantitative real-time polymerase chain reaction

The qRT-PCR analysis was completed using the SYBR Green kit (Bio-Rad, Hercules, CA, USA) and a Bio-Rad iCycler iQ5 real-time detection system. The qRT-PCR primers were designed using Primer-BLAST (http://www.ncbi.nlm.nih.gov/). The efficiency of all primer pairs was <90% (Table S6). The qRT-PCR condition was as follows: 95°C for 5 min; 40 cycles at 95°C for 30 s, 62°C for 15 s, and 72°C for 45 s; 95°C for 1 min and 55°C for 1 min. At the end of each qRT-PCR experiment, a melt curve was generated to rule out the possibility of primer-dimer formation. The qRT-PCR analysis was completed with three biological replicates, each consisting of three technical replicates. The relative expression levels were calculated using SPB actin gene expression as the internal reference. Significant differences in the data for the control and treatment groups were determined based on a one-way analysis of variance (*p* < 0.05 and *p* < 0.01; Student's *t*-test, *n* = 3).

## Results

### Analyses of sequence assemblies

**Figure 1:**
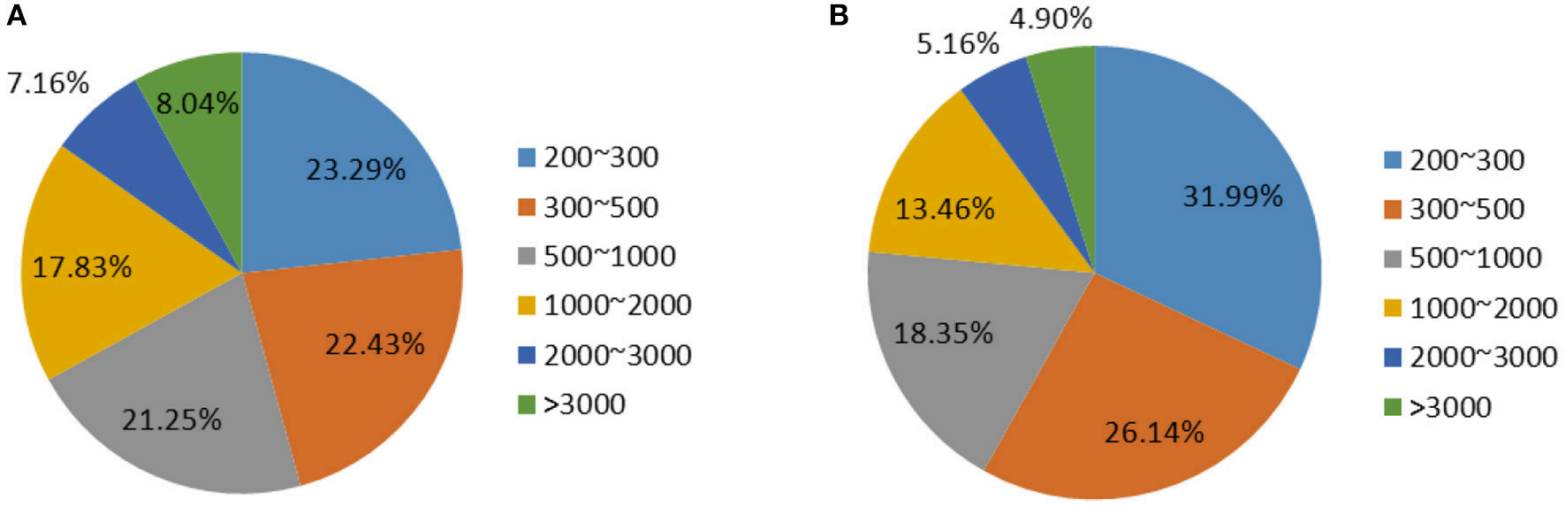
Overview of the SPB transcriptome sequencing and assembly. **(A)** Length distribution of transcripts. **(B)** Length distribution of Unigenes.

To compare the transcriptomes of SPBs fed with pods of transgenic dsSpbP0 and wild-type DN50 soybean plants, six SPB cDNA libraries were constructed for RNA-sequencing (RNA-seq) using the Illumina HiSeq 2000 system. After a quality check and the removal of rRNA sequences, 135,381,412 high-quality reads were assembled into 9,317,910 contigs using the Trinity *de novo* assembly program. The contigs were incorporated into 119,631 transcripts with an average length of 1096.91 bp (Tables S1, S2). After clustering and assembly analyses, 60,685 unigenes with a mean length of 846.20 bp were obtained, and the length distribution of the transcripts exhibited a similar trend to that of the unigenes (Figure 1).

### Sequence annotations

The obtained unigenes were annotated following a BLASTX search of various protein databases, including the Clusters of Orthologous Groups (COG), Gene Ontology (GO), Kyoto Encyclopedia of Genes and Genomes (KEGG), UniProt/Swiss-Prot, NCBI non-redundant (NR) databases. A total of 28,338 unigenes (46.69%) were annotated based on the COG, GO, KEGG, and NR databases (Table S3). The other 32,347 unigenes were un-annotated possibly because of the short sequence reads generated by the sequencing methodology.

**Figure 2:**
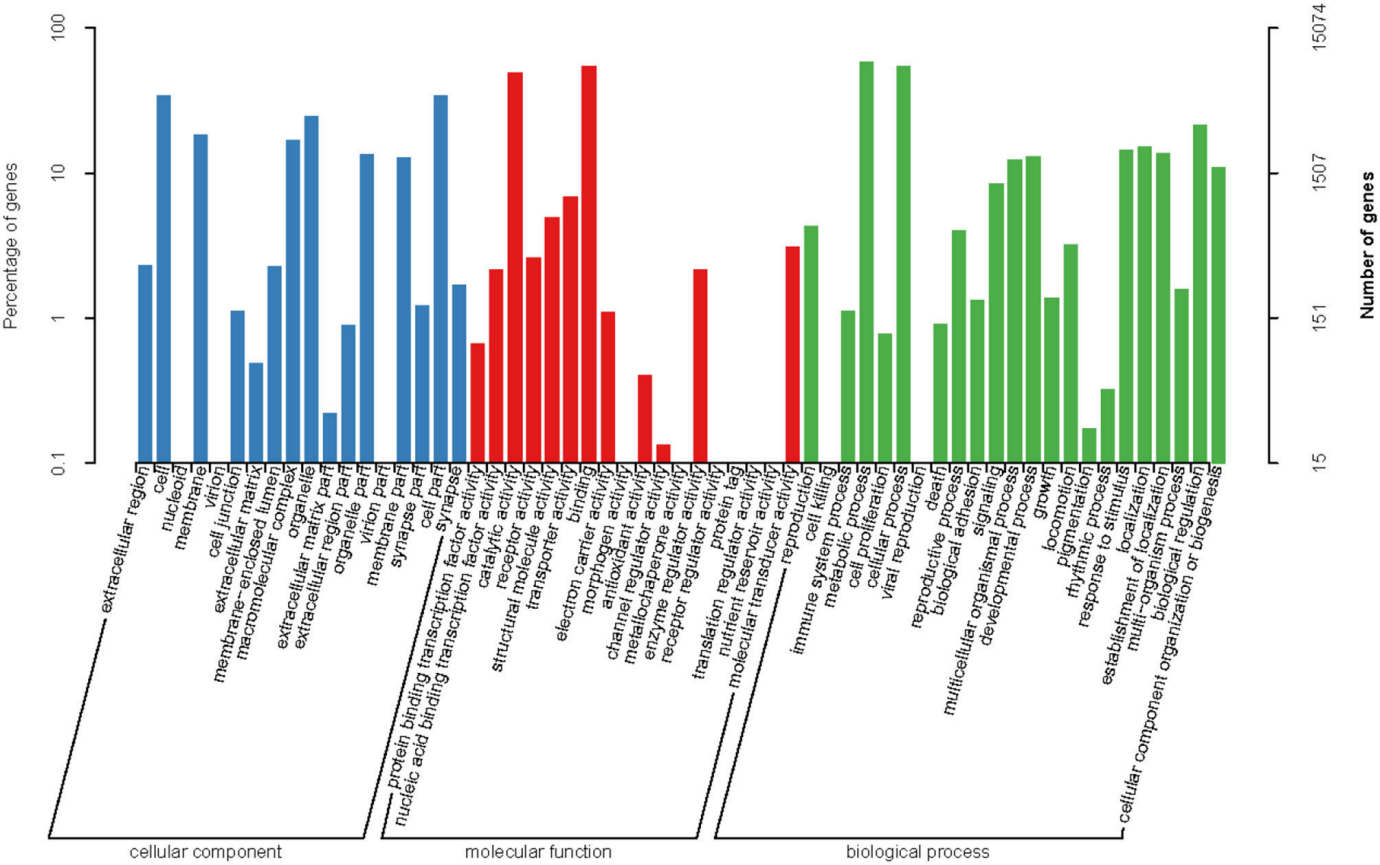
Functional annotations of assembled sequences based on gene ontology (GO) categories.

For the GO analysis, 15,079 unigenes were annotated with 80,333 GO functions based on sequence homology, with an average of 5.33 GO annotations per unigene. These included 24,774 annotations (30.83%) for cellular component, 19,208 annotations (23.91%) for molecular function, and 36.351 annotations (45.25%) for biological process (Figure 2). For the cellular component category, genes associated with the cell and cell parts were the most represented. For the molecular function category, most of the genes were related to binding and catalytic activity. Regarding the biological process genes, the most represented categories were metabolic process and cellular process.

**Figure 3:**
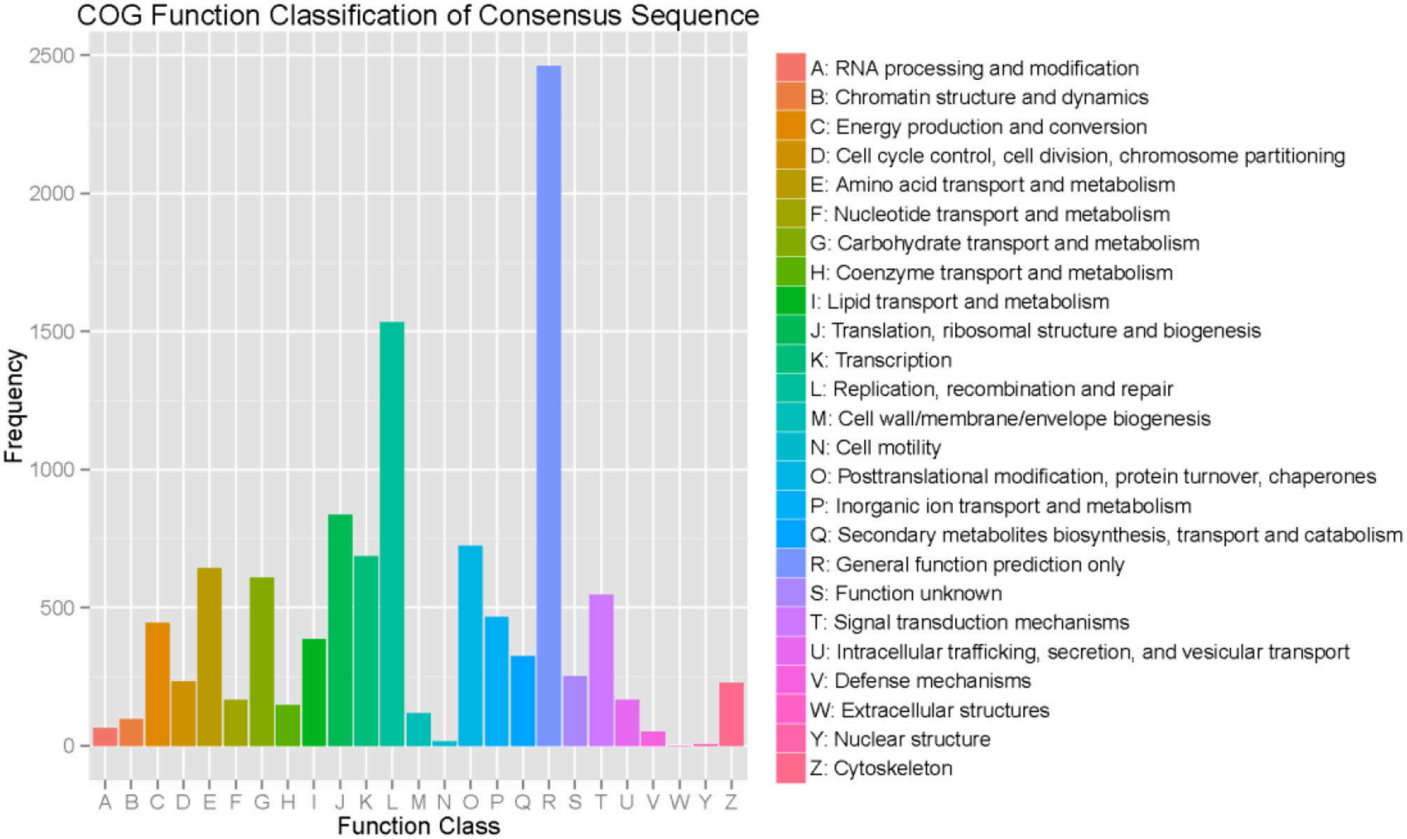
Functional classifications based on the Clusters of Orthologous Groups protein database.

Regarding the COG functional classification, 8,503 unigenes with hits in the NR database were grouped into 25 different functional classes (Figure 3). General function prediction represented the largest group, followed by replication, recombination and repair, translation, ribosomal structure, biogenesis, amino acid transport, and metabolism.

To identify the biochemical pathways active in the SPB, unigenes were analyzed using the KEGG annotation system. This process predicted a total of 216 pathways represented by 8,861 unigenes. The pathways with the most unigenes were related to the ribosome (ko03010, 395 unigenes, 4.45%), followed by RNA transport (ko03013, 361 unigenes, 4.077%), protein processing in endoplasmic reticulum (ko04141, 352 unigenes, 3.97%), spliceosome (ko03040, 351 unigenes, 3.96%), oxidative phosphorylation (ko00190, 315 unigenes, 3.55%), purine metabolism (ko00230, 216 unigenes, 2.43%), lysosome (ko04142, 197 unigenes, 2.22%), endocytosis (ko04144, 192 unigenes, 2.16%), ubiquitin-mediated proteolysis (ko04120, 186 unigenes, 2.09%) and RNA degradation (ko03018, 184 unigenes, 2.07%; Table S4). The pathways associated with RNA transport, endocytosis, and RNA degradation were important for in-depth analyses of SPB RNA-related genes.

### Identification of RNA interference-related genes in the soybean pod borer transcriptome

The sequences representing the genes involved in RNAi pathways were identified in the SPB transcriptome (Table S5).

#### Core RNA interference machinery genes

**Figure 4:**
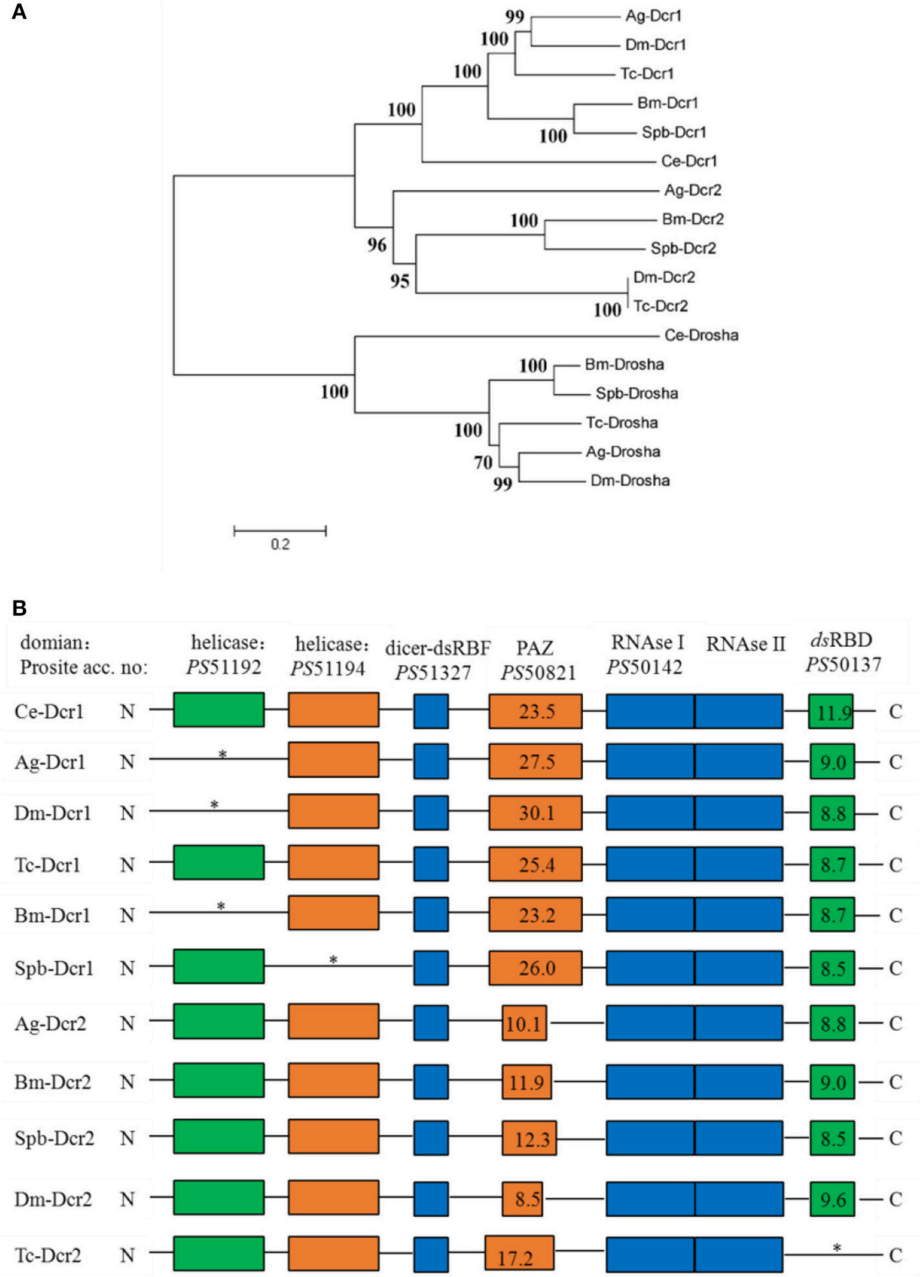
Phylogenetic and domain architecture analysis of Dicer proteins. **(A)** Neighbor-joining inferred phylogenetic relationships among insect Dicer proteins, including CeDcr-1 (NP_498761), AgDcr-1 (AAO73809), DmDcr-1 (NP_524453), BmDcr-1 (XP_004922366), TcDcr-1 (EFA11550), SpbDcr-1 (c79781), AgDcr-2 (XP_320248), BmDcr-2 (NP_001180543), SpbDcr-2 (c79492), DmDcr-2 (NP_23778.2), TcDcr-2 (NP_001107840), CeDrosha (O01326), AgDrosha (XP_555381), BmDrosha (XP_004928266), TcDrosha (XP_008199088), SpbDrosha (c79402). Bootstrap values (1,000 replicates) are shown next to the branches. **(B)** Domain architecture of Dicer proteins. Conserved domains and their ScanProsite profile hit scores are indicated. Ce, *Caenorhabditis elegans*, Bm, *Bombyx mori*; Dm, *Drosophila melanogaster*; Tc, *Tribolium castaneum*; Ag, *Anopheles gambiae*; SPb, soybean pod borer.

**Figure 5:**
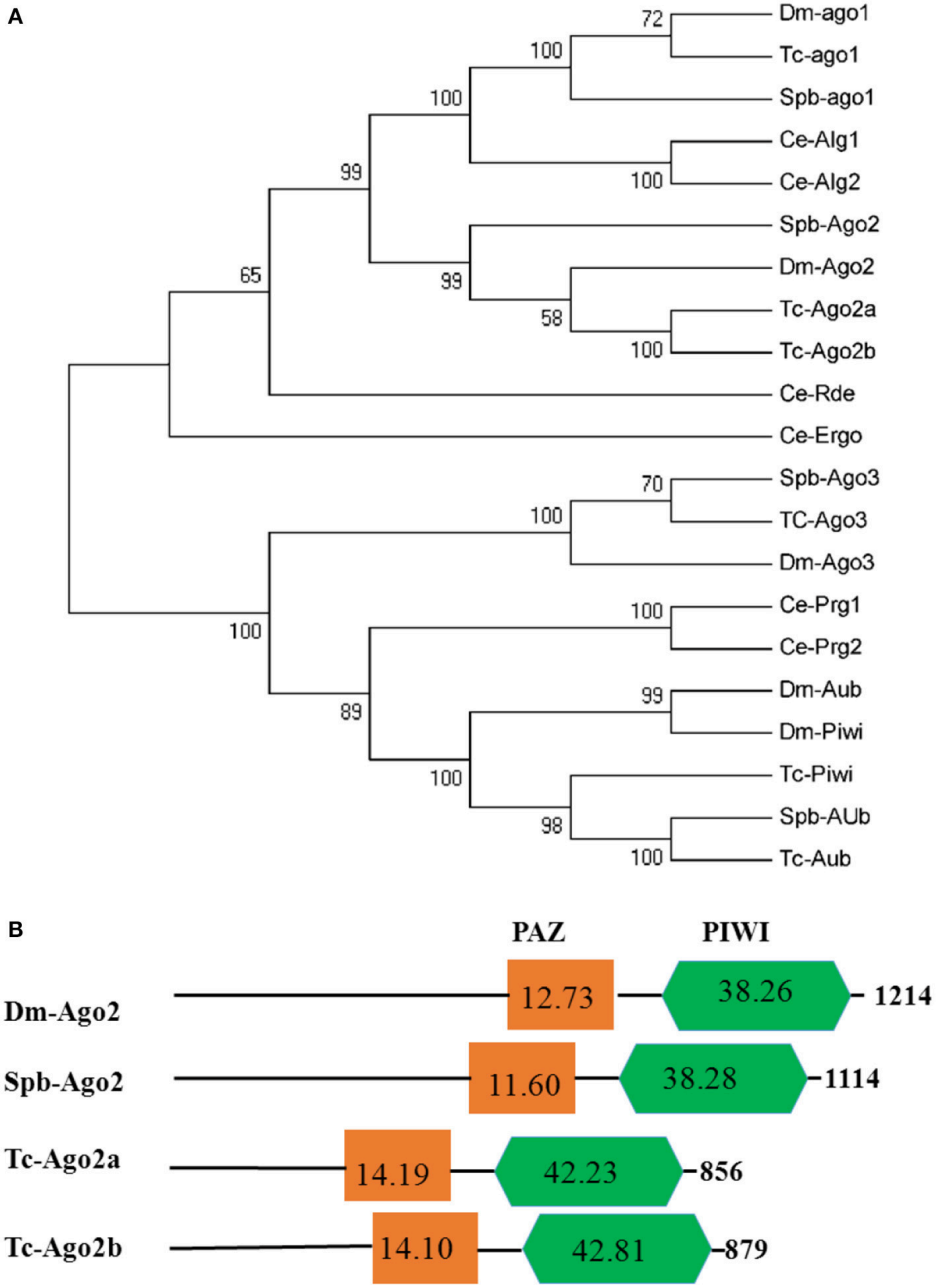
Phylogenetic and domain architecture analysis of Argonaute proteins. **(A)** Neighbor-joining inferred phylogenetic relationships among insect Argonaute proteins, including Dm-ago1 (NP_725341), Tc-ago1 (EFA09197), Spb-ago1 (C70013), CeAlg1 (CAA93496), CeAlg2 (CCD73271), Spb-ago2 (C65749), Dm-ago2 (NP_648775), Tc-ago2a (NP_001107842), Tc-ago2b (NP_0011078286651), Ce-Ergo (CCD62622), Ce-Rde (CAB05546), Spb-ago3 (C79403), Tc-ago3 (XP_008192425), Dm-ago3 (ABO27430), Ce-Prg1 (NP_492121), Ce-Prg2 (NP_500994), Dm-Aub (CAA64320), Dm-Piwi (CAAD08705), Tc-Piwi (EFA07425), Spb-Aub (C63841), Tc-Aub (XM_008198081). Bootstrap values (1,000 replicates) are provided next to the branches. **(B)** Domain architecture of Argonaute2 proteins. The PAZ and PIWI domains are indicated. Ce, *Caenorhabditis elegans*; Bm, *Bombyx mori*; Dm, *Drosophila melanogaster*; Tc, *Tribolium castaneum*; SPb, soybean pod borer.

Dicer, Argonaute, and double-stranded RNA-binding proteins (dsRBPs) are core components in the RNAi machinery. Two Dicer genes and one Drosha gene were identified in the SPB transcriptome. One gene, Spb-Dcr1, clustered with Bm-Dcr1. The second SPB Dicer clustered with Bm-Dcr2 (Figure 4A). Meanwhile, the ScanProsite search indicated that Spb-Dcr2 contains the helicase and PAZ domains, with low scores for the PAZ domain (Figure 4B), making Spb-Dcr2 similar to Bm-Dcr2. Specific dsRNA-binding proteins associated with Dicer load sRNA molecules into the RISC. Orthologs of *T. castaneum* Loquacious, R2D2, and Pasha were found in the SPB transcriptome (Figure S1A). An analysis of the domains on the putative Spb-R2D2 protein with ScanProsite uncovered the presence of three specific dsRNA-binding domains (Figure S1B). Argonaute proteins are important components of the RISC that affect gene silencing mediated by small non-coding RNA. Four Argonaute genes were identified in the SPB transcriptome. Phylogenetic analyses confirmed that Spb-Ago1 was included in the miRNA class of Argonaute proteins (Ago1), while Spb-Ago2 was an siRNA class Argonaute protein. Additionally, Spb-Ago3 and Spb-Aub were clustered with the piRNA class Argonaute proteins (Figure 5A). The PAZ and PIWI domains were identified in Spb-Ago2 as well as in homologous proteins of other insects (Figure 5B). These two domains are highly conserved in insects. The PAZ domain binds siRNA, while the PIWI domain exhibits RNase activity. The results of the phylogenetic analysis suggest that SPB-Ago2 is most closely related to the *D. melanogaster* Ago2, among the examined sequences.

#### Candidate dsRNA uptake genes

**Figure 6:**
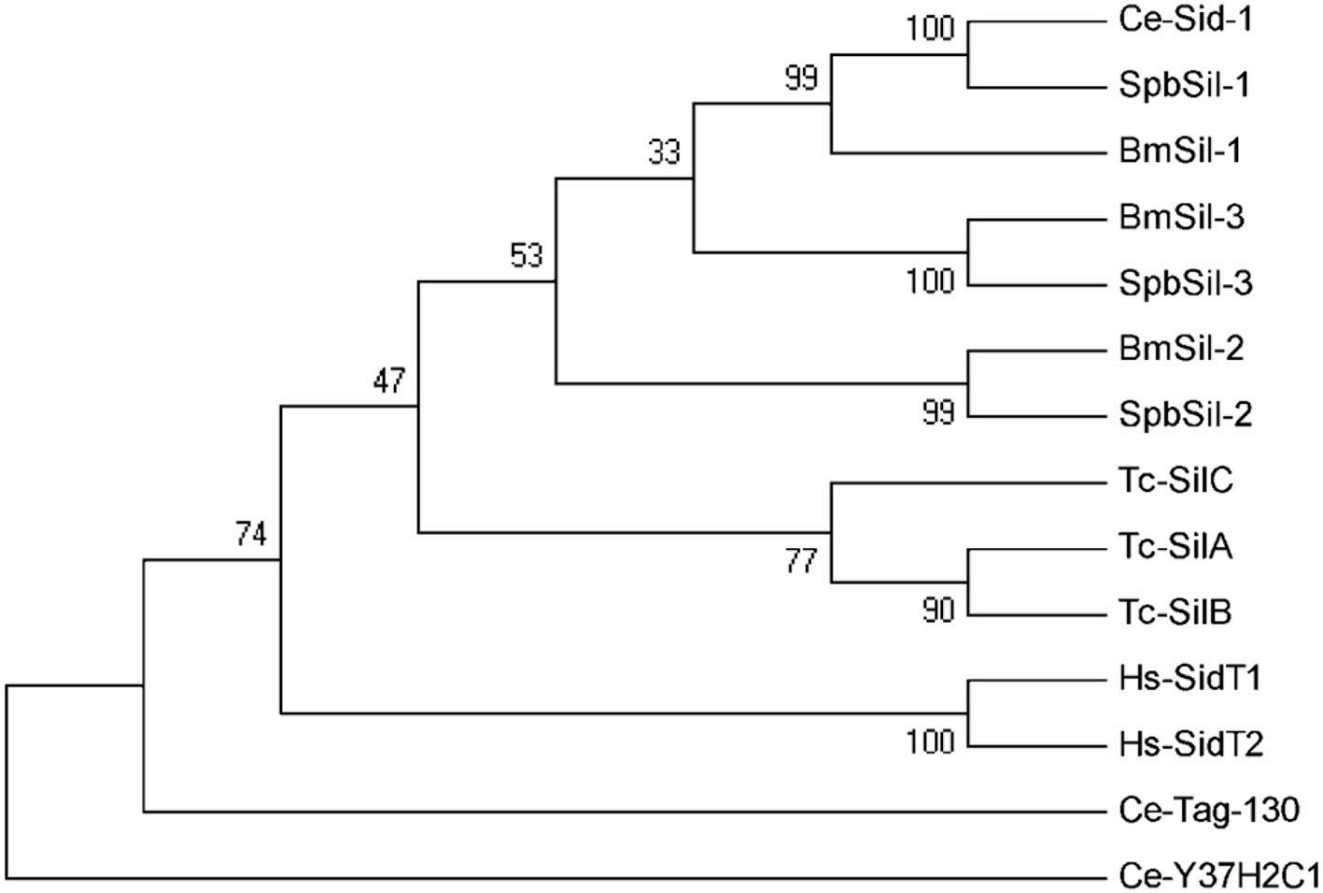
Phylogenetic analysis of sid-1-like (sil) proteins. Neighbor-joining inferred phylogenetic relationships among insect sil proteins, including Ce-Sid-1 (AAL78657), Spbsil-1 (C77135), Bmsil-1 (BAF95805), Bmsil-2 (BAF95807), Spbsil-2 (C79601), Bmsil-3 (BAF95806), Spbsil-3 (C79695), Tc-silA (NP_001099012), Tc-silB (NP_ 001103253), Tc-silC (NP_ 001099128), Hs-SidT1 (EAW79633), Hs-SidT2 (EAW67296), Ce-Tag-130 (ABU75284), Cel-Y37H2C1CAA19499). Bootstrap values (1,000 replicates) are provided next to the branches. Ce, *Caenorhabditis elegans*, Bm, *Bombyx mori*; Dm, *Drosophila melanogaster*; Tc, *Tribolium castaneum*; Hs, *Homo sapiens*; Spb, soybean pod borer.

Insect pests take up dsRNA using two different pathways, namely the systemic RNAi-deficient (SID) transmembrane channel-mediated pathway and the receptor-mediated endocytic pathway. In this study, the SPB transcriptome was screened to detect homologs of genes involved in both pathways. Three full-length *sil* genes were identified in the SPB transcriptome. Spb-sil1 was orthologous to Ce-sid1 and Bm-sil1, Spb-sil2 was orthologs of Bm-sil2, and Spb-sil3 was orthologs to Bm-sil3 (Figure 6). According to the TMHMM Server 2.0, three SPBsil proteins contained a long amino terminal extracellular domain followed by 11 transmembrane regions. These regions were highly similar among the sid-1 homologs (Figure S2). Additional domains or motifs were not identified by a ScanProsite search.

**Figure 7:**
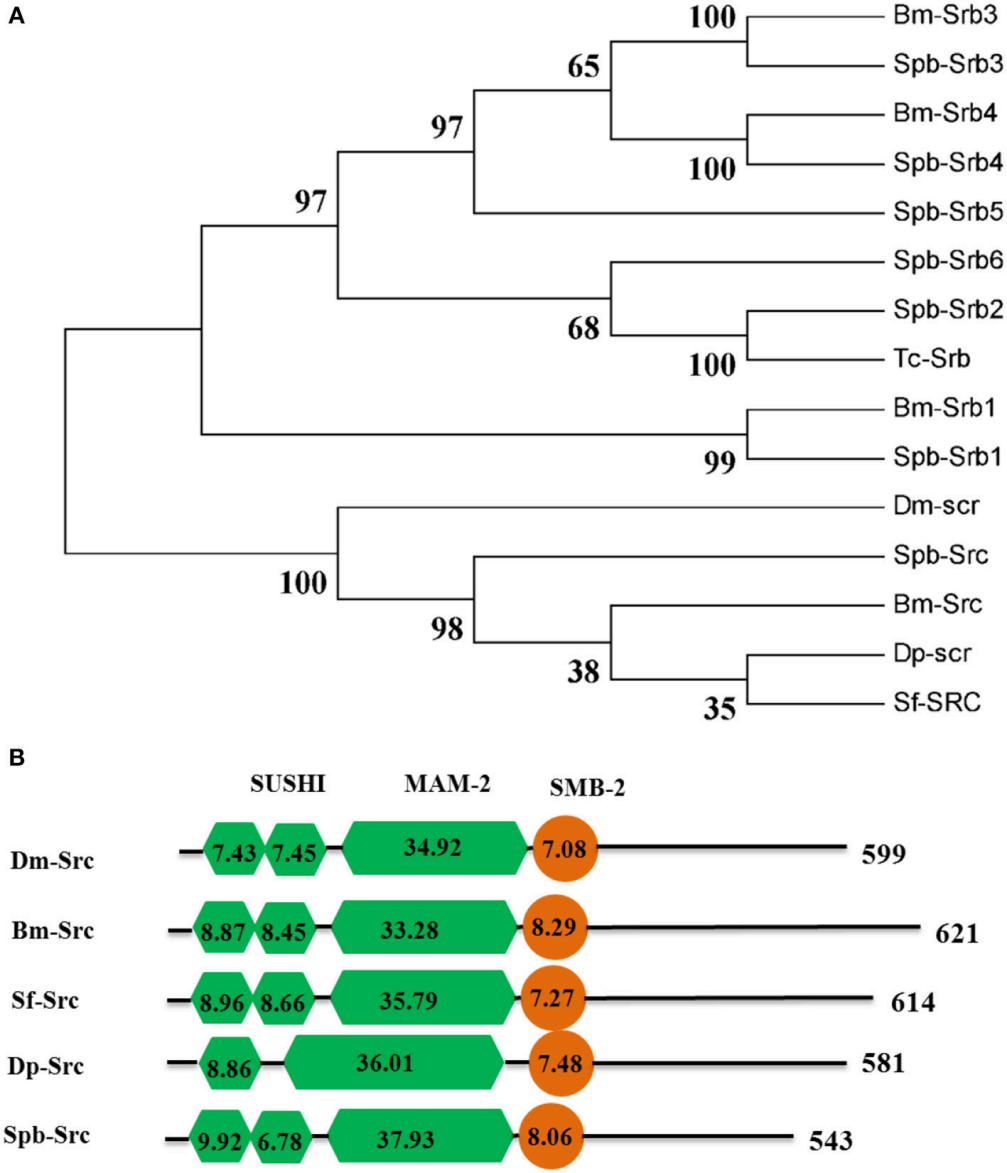
Phylogenetic and domain architecture analysis of scavenger receptor proteins. **(A)** Neighbor-joining inferred phylogenetic relationships among insect scavenger receptor proteins, including BmSrb3 (NP_001164650), SpbSrb3 (C74700), BmSrb4 (NP_001164651), SpbSrb4(C66328), SpbSrb5 (C61934), SpbSrb6 (C76572), SpbSrb2 (C78248), BmSrb1 (XP_971917), SpbSrb1 (C75587), TcaSrb (XP_001164151), DmSrc(AFF58551), BmSrc (NP_001128387), SfSrc (ABB9283), SpbSrc (C75587), DpSrc(EHJ6731). Bootstrap values (1,000 replicates) are provided next to the branches. **(B)** Domain architecture of scavenger receptor C proteins. The SUSHI, MAM-2, and SMB-2 domains are indicated. *Bombyx mori*; Dm, *Drosophila melanogaster*; Tc, *Tribolium castaneum*; Sf, *Spodoptera frugiperda*; Spb, soybean pod borer.

The dsRNA in the midgut lumen is taken up by the surrounding cells *via* endocytosis. Meanwhile, scavenger receptors function as dsRNA receptors during the endocytic process. Seven scavenger receptor genes were identified in the SPB transcriptome. Only one SPB homolog (Spb-Src) belonged to scavenger receptor class C, while the other six were class B scavenger receptors (Figure 7A). The three domains (i.e., SUSHI, MAM-2, and SMB-2) identified in Spb-Src were highly conserved in other Lepidoptera insects and in *D. melanogaster* (Figure 7B).

### Establishment of the RNAi-of-RNAi assay system to identify components of the soybean pod borer RNA interference pathway

**Figure 8:**
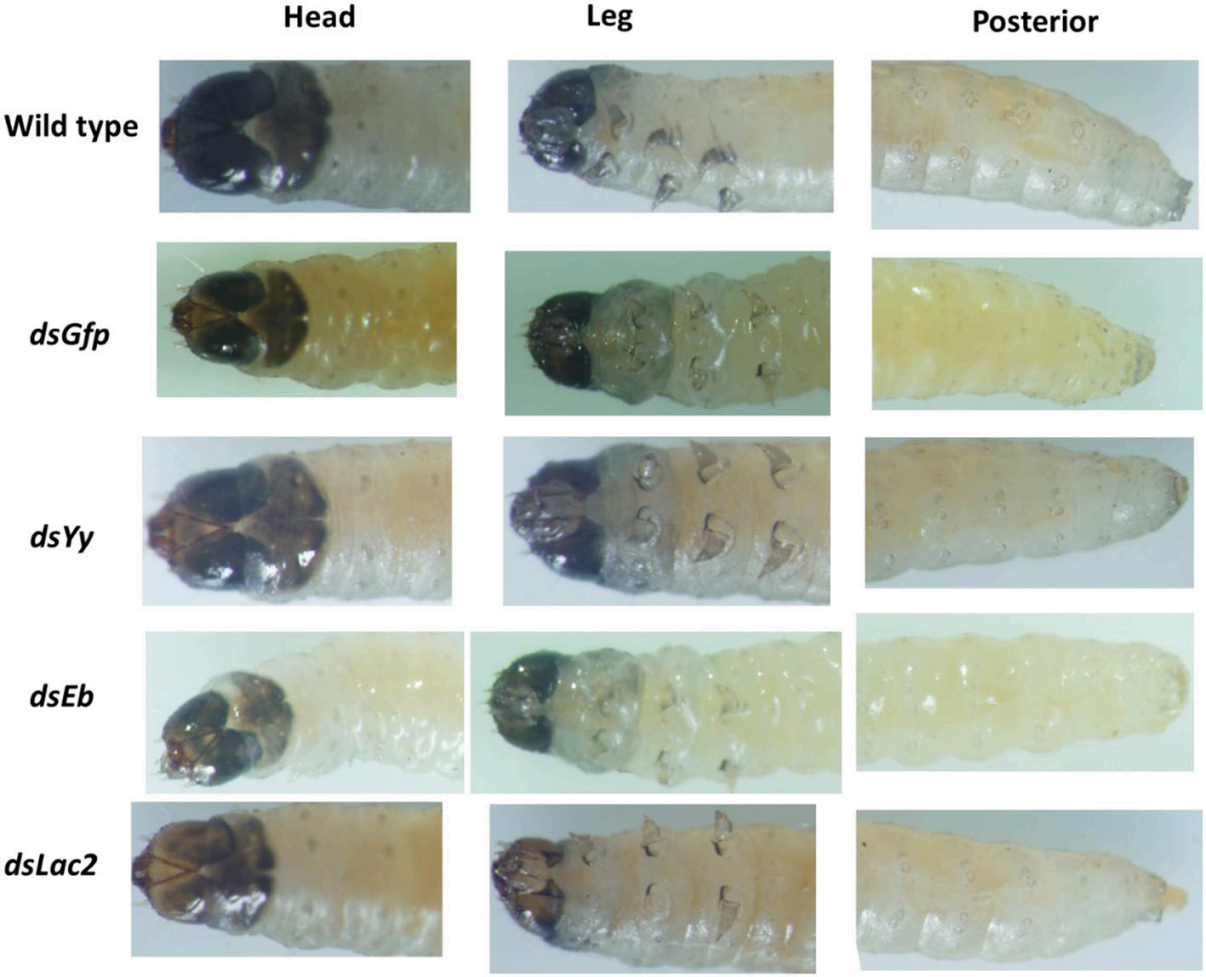
Phenotypes resulting from the *Lac2* feeding RNAi. *Lac2* RNAi affected the pigmentation of the larval head. The larvae were fed 5 ug/ml 750-bp dsRNA of Gfp,*Yellow-y* (*Yy*), *laccase-2* (*Lac2*), and *ebony* (*Eb*) gene separately.

*Yellow-y* (*Yy*), *laccase-2* (*Lac2*), and *ebony* (*Eb*) are the main pigmentation biosynthesis genes in insects. Loss-of-function or RNAi experiments involving these genes resulted in insects with decreased or increased pigmentation that did not affect larval mortality (Miyata et al., 2014). The SPB orthologs for *Yellow-y, laccase-2*, and *ebony* were identified using the transcriptome data. The first-instar larvae (1 day after hatching) were fed 750-bp dsRNA targeting these genes with an artificial diet (final dsRNA concentration: 5 μg/ml). The color of the larval bodies was analyzed after the first larval molt. The RNAi-mediated silencing of *Lac2* led to decreased black pigmentation in the head. In contrast, RNAi-based silencing of *ebony* and *yellow-y* did not influence pigmentation (Figure 8).

**Figure 9:**
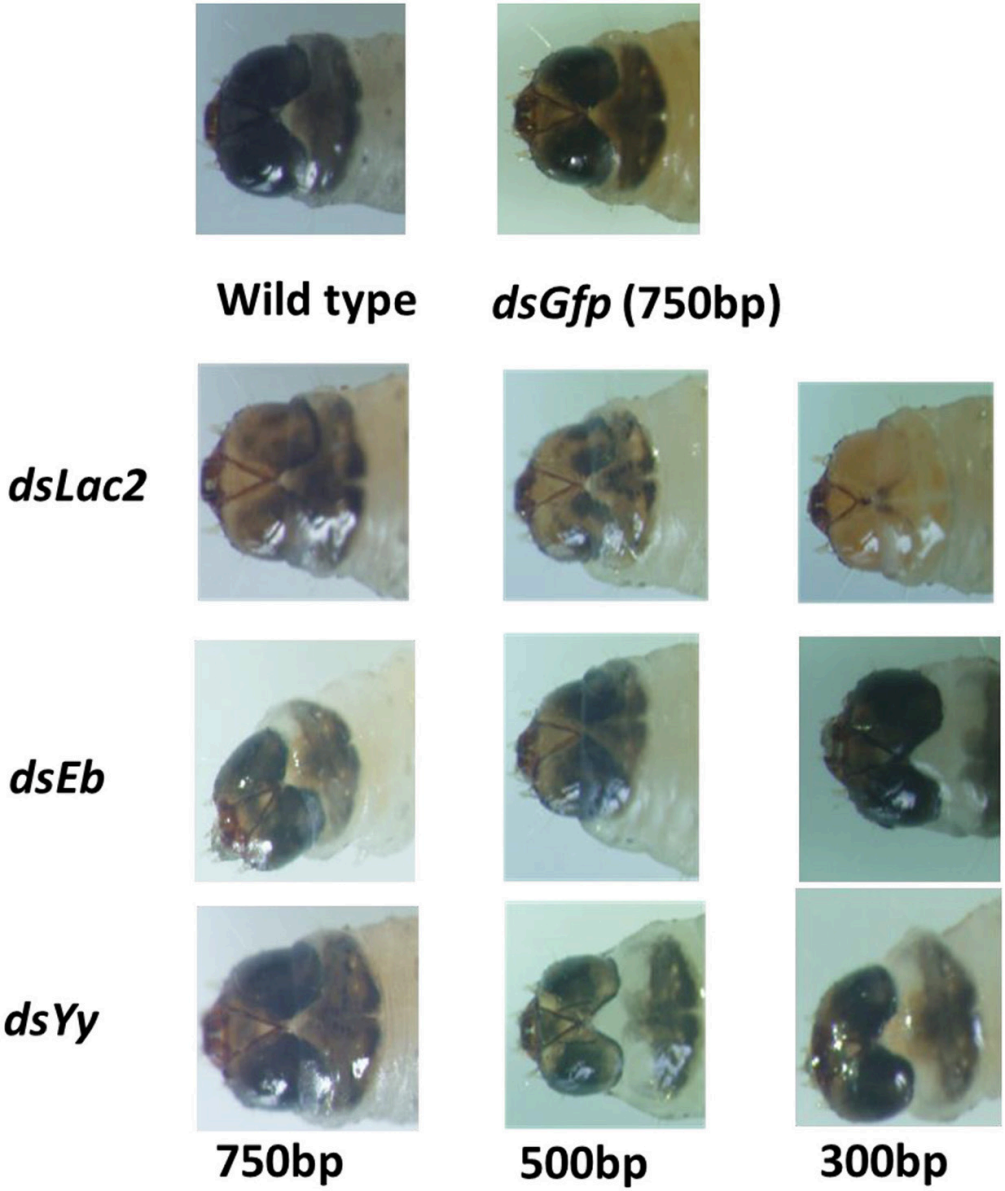
Effect of dsRNA length on feeding RNAi efficiency. Feeding 5 ug/ml 300 bp long of Lac2 dsRNA induced easily identifiable pigmentation defects in Lac2 RNAi.

**Figure 10:**
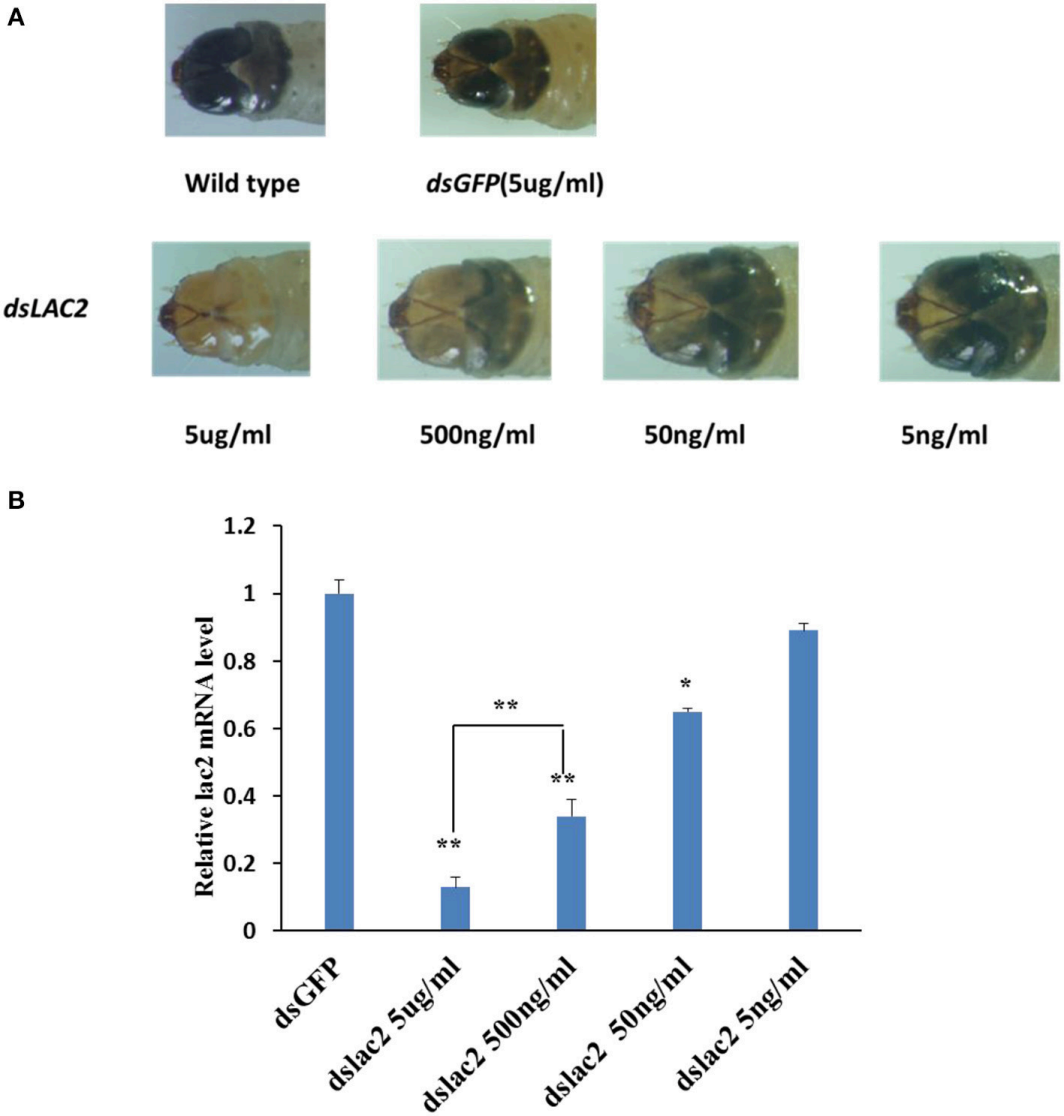
Effect of dsRNA does on feeding RNAi efficiency. **(A)** Lac2 RNAi with various doses of dsRNA. Feeding 500 ng/ml or more of dsRNA induced easily identifiable pigmentation defects in lac2 RNAi. **(B)** Relative expression level of lac2 by various doses of Lac2 dsRNA. Data were analyzed using Student's *t*-test (**p < 0.01, ^*^p < 0.05).

Different lengths of *Lac2* dsRNA were tested to evaluate whether dsRNA length affected the pigmentation defect. Of the tested lengths, 300 bp dsRNA molecules produced the expected Lac2 RNAi phenotype most prominently (Figure 9). Thus, 300 bp *Lac2* dsRNA was used to analyze the effects of dsRNA concentration on the pigmentation defect. Larvae fed with 5 μg/ml and 500 ng/ml dsRNA exhibited clear pigmentation defects (Figure 10A). The decrease in *Lac2* mRNA abundance was confirmed by quantitative real-time polymerase chain reaction (qRT-PCR; Figure 10B). Therefore, 5 μg/ml 300 bp dsRNA was used to evaluate pigmentation when *lac2* was used as the marker.

**Figure 11:**
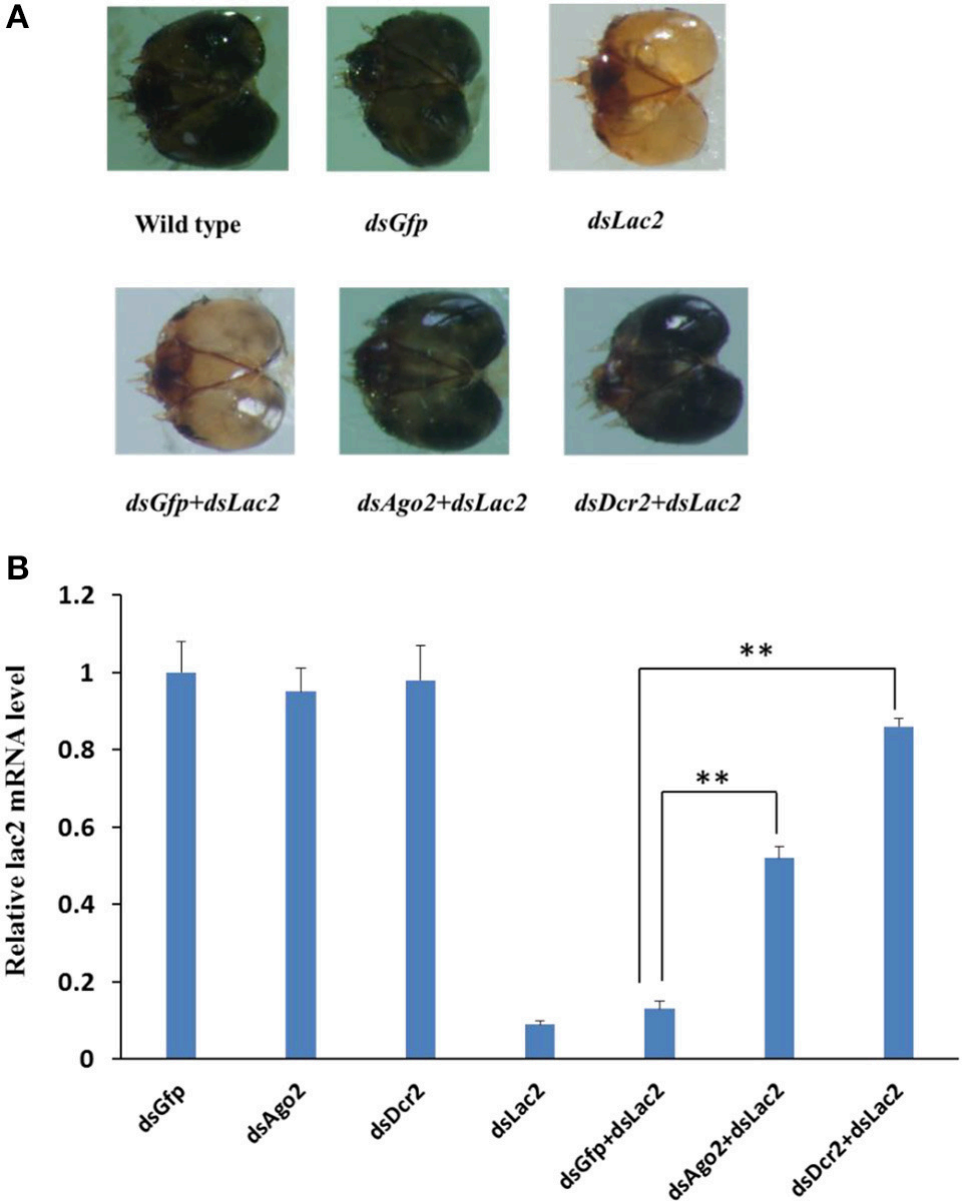
*Dcr2* and *Ago2* RNAi efficiently suppressed the *Lac2* RNAi. **(A)** Heads of a wild-type larva as well as larvae fed *Gfp* dsRNA, *Lac2* dsRNA, *Gfp* dsRNA + *Lac2* dsRNA, *Ago2* dsRNA + *Lac2* dsRNA, or *Dcr2* dsRNA + *Lac2* dsRNA. Both *Ago2* and *Dcr2* RNAi, but not *Gfp* RNAi, suppressed the *Lac2* RNAi phenotype. **(B)** Relative *lac2* expression level based on a qRT-PCR. The *Lac2* expression level was higher in larvae fed *Ago2* dsRNA or *Dcr2* dsRNA than in control larvae (i.e., fed *Gfp*dsRNA + *Lac2* dsRNA). Data were analyzed using Student's *t*-test (**p < 0.01).

The assay system was first tested with *Ago2* and *Dcr2*, which were core RNAi components in the insect siRNA pathway. The *Dcr2* and *Ago2* expression levels decreased by 78% and 65% in the first-instar larvae fed on dsRNA (5 μg/ml each) for 6 days. The first RNAi treatment involved 300 bp *Dcr2* and *Ago2* dsRNA, while the second RNAi treatment consisted of 300 bp *Lac2* dsRNA (5 μg/ml each). The specific suppression of *Dcr2* and *Ago2* expression was analyzed by qRT-PCR. The second RNAi effect was specifically suppressed by *Ago2* RNAi (by 52%) and *Dcr2* RNAi (by 86%), but not by the negative control green fluorescent protein (*GFP*) dsRNA (by 12%; Figure 11). The results indicated that the RNAi-of-RNAi system could be used to evaluate the SPB RNAi pathway genes.

### Involvement of *Sil* genes in dsRNA uptake

**Figure 12:**
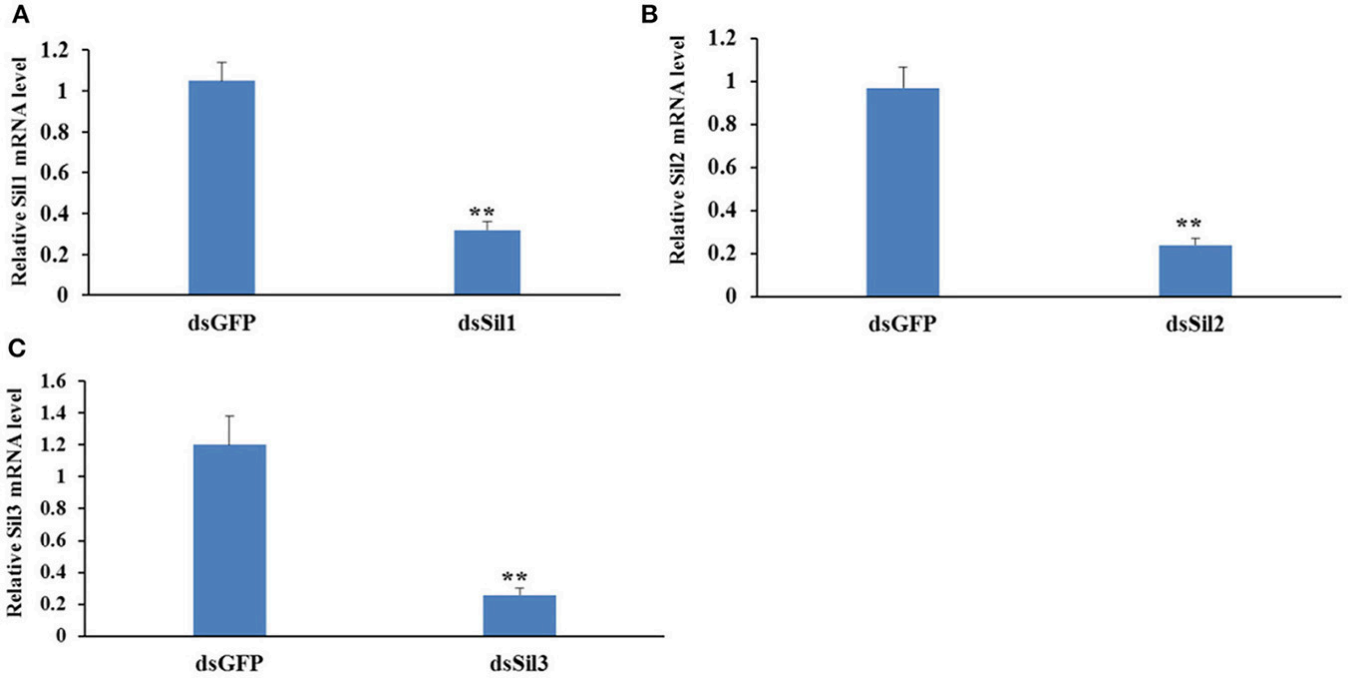
Relative expression level of *Sil* genes analyzed by qPCR, the first larvae were fed 5 ug/ml dsRNA of *Sil* genes separately. **(A)** Relative expression level of *Sil1* gene, fed on dsSil1 resulted in a silencing 73% for Sil1. **(B)** Relative expression level of *Sil2* gene, fed on dsSil2 resulted in a silencing 69% for *Sil2*. **(C)** Relative expression level of *Sil3* gene, fed on dsSil3 resulted in a silencing 78% for *Sil3* (**p < 0.01).

**Figure 13:**
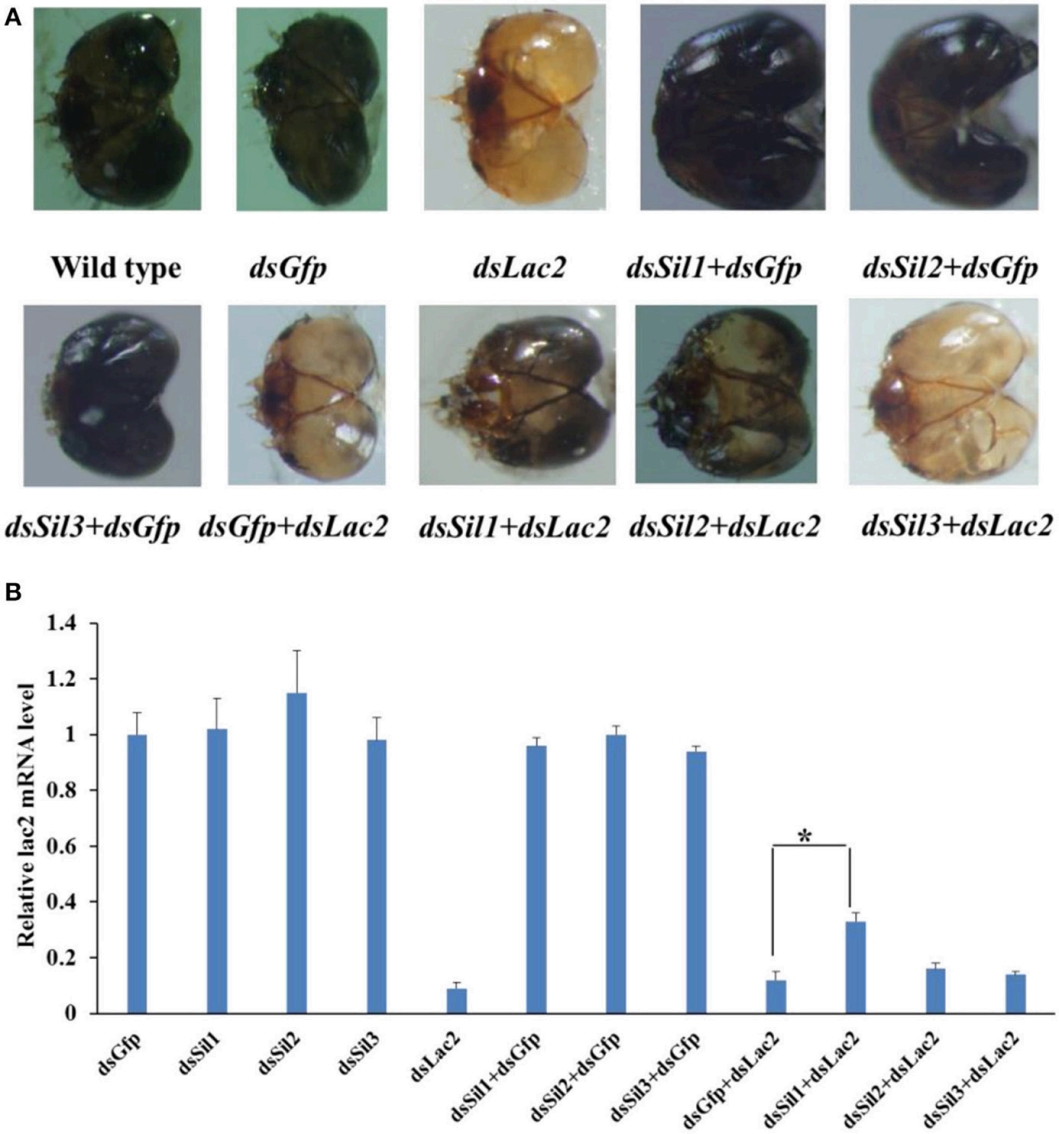
*Sil* RNAi efficiently suppressed the *Lac2* RNAi. **(A)** Heads of a wild-type larvae as well as larvae fed *Gfp* dsRNA, *Lac2* dsRNA, *Sil1* dsRNA + *Gfp* dsRNA, *Sil2* dsRNA + *Gfp* dsRNA, *Sil3* dsRNA + *Gfp* dsRNA, *Gfp* dsRNA + *Lac2* dsRNA, *Sil1* dsRNA + *Lac2* dsRNA, *Sil2* dsRNA + *Lac2* dsRNA, or *Sil3* dsRNA + *Lac2* dsRNA. Both *Sil1* and *Sil2* RNAi suppressed the *Lac2* RNAi phenotype. **(B)** Relative *Lac2* expression level based on a qRT-PCR. The *Lac2* expression level was higher in larvae fed *Sil1* dsRNA+ *Lac2* dsRNA than in control larvae (i.e., fed *Gfp* dsRNA + *Lac2* dsRNA). Data were analyzed using Student's *t*-test (*p < 0.05).

The expression level of *Spbsil1, Spbsil2*, and *Spbsil3* were similar in larvae (1.05 ± 0.09, 0.97 ± 0.06, and 1.2 ± 0.18, respectively). Identical dose of dsRNA (5 μg/ml) resulted in the silencing of 73, 69, and 78% of the *Spbsil1, SpbSil2*, and *SpbSil3* expression (Figure 12). Therefore, the three Spb *sil* genes were tested in our RNAi-of-RNAi system. In contrast to the effects of *Gfp* dsRNA and *Spbsil* **dsRNA** + *Gfp* dsRNA(negative control), the silencing of *Spbsil-1* decreased the RNAi efficiency of *Lac2* dsRNA. The relative *Lac2* mRNA level was higher for larvae expressing *sil1* (33% of the mRNA level was detected) than for the larvae expressing *Spbsil-2* or *Spbsil-3*, in which 16 and 14% of the *Lac2* mRNA levels were detected, respectively, with no significant differences from the results of the *Gfp* dsRNA treatment (12% of the mRNA level was detected; Figure 13).

### Involvement of the endocytic pathway components in dsRNA uptake

**Figure 14:**
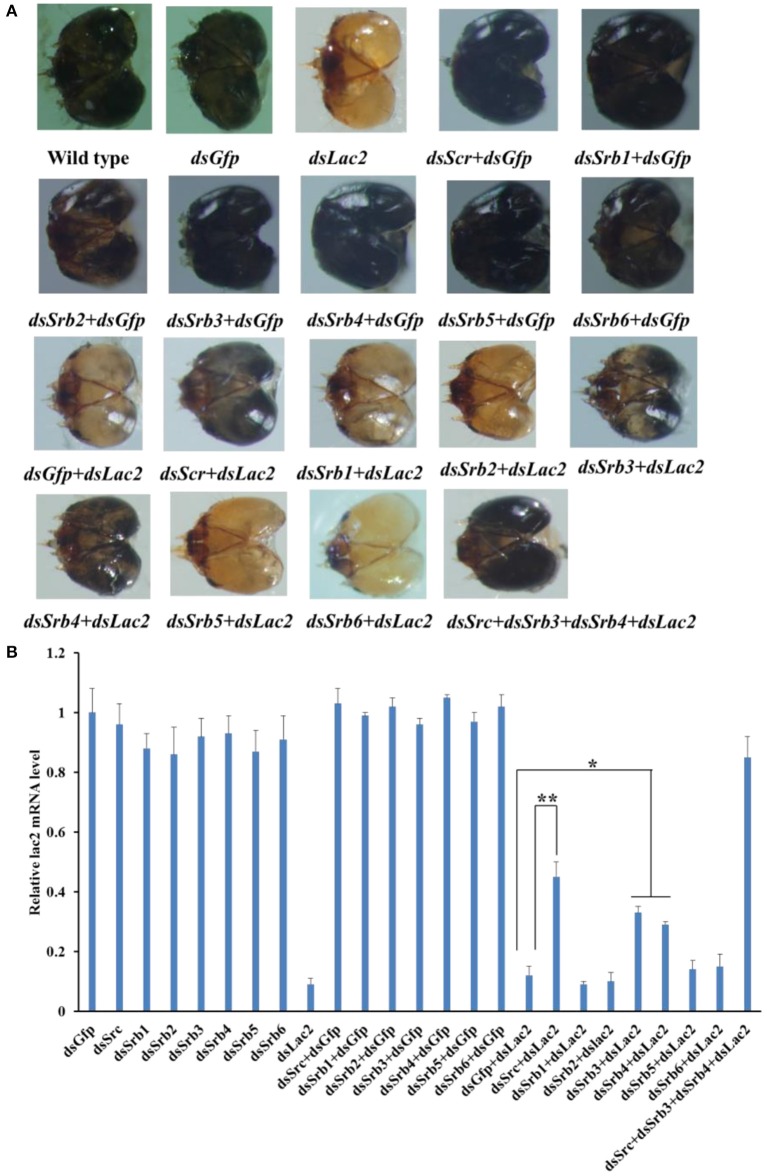
Involvement of scavenger receptors C and B (Src and Srb) in dsRNA uptake by the soybean pod borer. **(A)** Heads of a wild-type larva as well as larvae fed *Gfp* dsRNA, *Lac2* dsRNA, *Src* dsRNA + *Gfp*dsRNA, *Srb* dsRNA + *Gfp*dsRNA, *Gfp*dsRNA + *Lac2* dsRNA, *Src* dsRNA + *Lac2* dsRNA, or *Srb* dsRNA + *Lac2* dsRNA. Treatments with 5 μg/ml *Src, Srb3*, or *Srb4* dsRNA suppressed the *Lac2* RNAi. A simultaneous treatment with 5 μg/ml *Src, Srb3*, and *Srb4* resulted in a complete rescue of the phenotype. **(B)** Relative *Lac2* expression level based on a qRT-PCR. All data were normalized against the *Lac2* expression levels in the control larvae fed *Gfp* dsRNA + *Lac2* dsRNA. Data were analyzed using Student's *t*-test (**p < 0.01; ^*^p < 0.05).

Seven scavenger receptor genes of the endocytic pathway were tested in our RNAi-of-RNAi system. For scavenger receptor class C (Src), a phenotypic rescue of 45% was detected when *Src* expression was silenced by 82%. Additionally, 33 and 29% of the expected phenotype were rescued when *Srb3* (i.e., scavenger receptor class B) was silenced by 79% and *Srb4* was silenced by 85%, respectively. The suppression of the *Lac2* RNAi phenotype by *Src, Srb3*, and *Srb4* RNAi was robust, and led to a significan decrease in head pigmentation loss, with *Srb3* and *Srb4* RNAi having a smaller effect than *Src* RNAi. The silencing of *Lac2* was not recovered by other *Srb* genes, namely *Srb1, Srb2, Srb5*, and *Srb6*. To clarify the relationships among the three scavenger receptor genes, larvae were simultaneously fed *Src, Srb3*, and *Srb4* dsRNA. A near-complete phenotypic recovery (85%) was observed, which was greater than the effects of the silencing of individual scavenger receptor genes (Figure 14).

## Discussion

The SPB genome has not been sequenced, and the available sequence information is very limited, despite the fact that this pest is a major threat to soybean production in Asia (Wang et al., 2014). In this study, 119,631 transcripts from SPB larvae were generated and assembled into 60,685 unigenes. Although we focused on RNAi, our transcriptome data enabled the rapid discovery of diverse SPB candidate genes. Moreover, most representative unigenes were mapped to specific pathways of the KEGG database, including those related to the ribosome, RNA transport, and protein processing in the endoplasmic reticulum. This information might be useful for future analyses of the molecular mechanisms of transgenic soybean plants that express the dsRNA of the SPB ribosomal protein P0 gene (*SpbP0*) to enhance pest resistance (Meng et al., 2017b).

All core RNAi machinery-related genes were detected in the SPB transcriptome, often encoding full-length open reading frames (Table S5), implying that the SPB larvae contain functional miRNA, siRNA, and piRNA pathways. Additionally, the genes encoding SpbDcr2 and SpbAgo2 were more highly expressed in SPB larvae fed *SpbP0* dsRNA-expressing transgenic soybean pods than in the control larvae (Table S4). The main domains of the typical Dcr2 and Ago2 proteins were conserved in the corresponding SPB proteins (Kim et al., 2006; Sinha et al., 2015; Azlan et al., 2016). Our RNAi assay revealed that the reporter gene *Lac2* RNAi could be specifically suppressed by *Ago2* (52%) and *Dcr2* RNAi (86%), indicating that SpbDcr2 and SpbAgo2 are important for the SPB RNAi pathways. Furthermore, we observed that SpbDcr2 mediated the initial efficiency of RNAi, which was consistent with the findings of previous studies involving *T. castaneum, C. elegans*, and *Drosophila melanogaster* (Keeting et al., 2001; Lee et al., 2004).

In addition to these core genes, many other factors are known to affect the efficiency of RNAi (Tables S4; Taning et al., 2016). The *Lac2* expression levels decreased considerably, resulting in a lack of pigmentation in the heads of larvae fed *Lac2* dsRNA (Figure 8). Our results suggested that SPB larval cells took up dsRNA from the environment, and the siRNA was transmitted throughout the insect body. Similar results have been reported for other SPB RNAi studies (Meng et al., 2017a,b). Thus, the SPB exhibits a strong and systemic RNAi effect. The efficient uptake of dsRNA by cells and/or a lack of (or limited) dsRNA degradation might be primarily responsible for the robustness of the SPB systemic RNAi processes (Garbutt et al., 2013). The evolutionarily conserved Eri-1 protein, which was involved in intracellular siRNA degradation, contains the SAP/SAF-box and DEDDy family exonuclease domains (Kupsco et al., 2006). However, the homolog of *Eri-1* in the SPB transcriptome was not detected. The four other nuclease genes identified in the SPB transcriptome that were considered to be associated with RNAi-related activities (Table S3) comprised two paralogs of the *egalitarian* gene (Dienstbier et al., 2009), one homolog of an *oligoribonuclease* gene (Mwaengo and Lawrence, 2003), and one homolog of a snRNA-degrading nuclease gene (Yang et al., 2009). However, the proteins encoded by these genes lacked the SAP/SAF-box domain, and only the oligoribonuclease contained a DEDDy family exonuclease. This result suggested that these nucleases might have functions beyond the degradation of dsRNA in the SPB. Additionally, the efficient uptake of dsRNA by cells appeared to be the major factor responsible for the SPB systemic RNAi responses.

Although the involvement of the *sil* genes in the uptake of dsRNA have been demonstrated in *D. virgifera* and *L. decemlineata* (Miyata et al., 2014; Cappelle et al., 2016), it is unclear whether the orthologs of SID are associated with RNAi in moths (Xu et al., 2016). We detected three distinct *sil* genes, similar to the earlier studies of *Bombyx mori* and *Spodoptera litura* (Huvenne and Smagghe, 2010; Gong et al., 2015). These *sil* genes encoded the proteins with 11 transmembrane regions at the N-terminal end, similar to the *C. elegans* sid1 and tag10 proteins. Moreover, Spbsil1 contained an additional transmembrane domain at the C-terminus (Figure S2). The phylogenetic tree also revealed that Spbsil1 clustered with the *C. elegans* sid1 protein, whereas Spbsil2 and Spbsil3 were grouped with the *B. mori* sil sequences (Figure 6). In the RNAi-of-RNAi assay, the *Lac2* RNAi phenotype was rescued only when *SPBsil-1* expression was silenced, implying that SPBsil-1 is involved in the uptake of dsRNA, similar to the *C. elegans* sid1 protein.

Regarding endocytosis, silencing the genes encoding *Src, Srb3*, or *Srb4* significantly affected the silencing of the reporter gene, implying that scavenger receptor-mediated endocytosis influences the SPB systemic RNAi. Furthermore, a combination of *Src, Srb3*, and *Srb4* RNAi almost completely rescued the reporter gene RNAi response. The association between Src and dsRNA uptake has been reported for the *Drosophila* S2 cell line (Ulvila et al., 2006). The Srb members are physiologically relevant high-density lipophorin receptors. Lipophorins reportedly adhere to dsRNA in *B. mori* and desert locust hemolymphs (Duressa et al., 2014; Yang et al., 2016). Partial dsRNA molecules might adhere to lipophorins in the hemolymph. Additionally, the class B scavenger receptor of H. longicornis ticks (*HlSRB*) functioned in the uptake of exogenous dsRNAs in ticks (Aung et al., 2011). A more comprehensive characterization of this interaction might provide new insights into the associated pathway.

Scavenger receptor-mediated endocytosis and Sid-1-like proteins are involved in SPB cellular dsRNA uptake. The more robust systemic RNAi response associated with the scavenger receptor endocytosis-related genes. However, the suppression of the reporter gene RNAi by *Spbsil1* RNAi was not robust could be due to the fact that the silencing efficiency of sil1 *Spbsil1* was not 100%. These are knockdown experiments, rather than knockout experiments. Furthermore, protein half-life and expression-related differences, which could also have an impact on phenotypical effects (Hinas et al., 2012; Cappelle et al., 2016). Additional experiments are needed to elucidate the relative contribution of each pathway.

## Ethics statement

The SPB colony and plant materials were collected from the Key Laboratory of Soybean Biology of the Chinese Education Ministry. The collection and usage of samples followed the international, national, and/or institutional guidelines.

## Author contributions

FM and WL: Designed the experiments; FM, MY, and YL: Conducted the molecular studies; TL and ZW: Grew the plants; XL, ZW, GW, and XJ: Cultured the SPB; FM, YL, and WL: Wrote the first draft of the manuscript; YL and MY: Completed the statistical analyses. All authors read and approved the final manuscript.

### Conflict of interest statement

The authors declare that the research was conducted in the absence of any commercial or financial relationships that could be construed as a potential conflict of interest.

## Abbreviations

SPB: soybean pod borer
RNAi: RNA interference
SID: systemic RNA interference deficient
Sil: sid-like
SRC: scavenger receptor class C
SRB: scavenger receptor class B
Spbp0: soybean pod borer ribosomal protein P0
GFP: green fluorescent protein.
