# Supplementary material for: Functional Analysis of RNA Interference-Related Soybean Pod Borer (Lepidoptera) Genes Based on Transcriptome Sequences
Source: Front Physiol. 2018 May 3;9:383. doi: 10.3389/fphys.2018.00383 (PMC5943558; doi:10.3389/fphys.2018.00383)
Supplement: Supplementary file 1 [file Table_1.DOCX]

**Table S1** Summary of Illumina sequencing reads

| Sample | Raw reads | Total nucleotides(bp) | Cleaning reads | Q30 (%) | GC (%) |
| --- | --- | --- | --- | --- | --- |
| ck1 | 29,857,163 | 6030535897 | 22,576,866 | 92.51 | 51.01 |
| ck2 | 30,323,680 | 6,124,803,693 | 23,169,277 | 92.6 | 50.61 |
| ck3 | 29,222,946 | 5,902,489,430 | 22,233,355 | 92.78 | 49.16 |
| R-1 | 27,803,736 | 5,615,857,127 | 20,723,567 | 92.6 | 49.68 |
| R-3 | 31,096,491 | 6,280,936,080 | 23,992,343 | 92.74 | 48.91 |
| R-4 | 30,112,313 | 6,082,105,372 | 22,686,004 | 92.43 | 50.9 |
