# Supplementary material for: Functional Analysis of RNA Interference-Related Soybean Pod Borer (Lepidoptera) Genes Based on Transcriptome Sequences
Source: Front Physiol. 2018 May 3;9:383. doi: 10.3389/fphys.2018.00383 (PMC5943558; doi:10.3389/fphys.2018.00383)
Supplement: Supplementary file 2 [file Table_2.DOCX]

**Table S2** Summary of transcriptome assembly for SPB

| Length_span | Transcripts | Unigenes |
| --- | --- | --- |
| 0~200 | 0 | 0 |
| 200~300 | 27853 | 19414 |
| 300~500 | 26837 | 15858 |
| 500~1000 | 25427 | 11159 |
| 1000~2000 | 21328 | 8160 |
| 2000~3000 | 8570 | 3129 |
| >3000 | 9616 | 2965 |
| Total number | 119631 | 60685 |
| Total length | 131224583 | 51351373 |
| N50 length | 2001 | 1589 |
| Mean length | 1096.911194 | 846.1955 |
