# Supplementary material for: Functional Analysis of RNA Interference-Related Soybean Pod Borer (Lepidoptera) Genes Based on Transcriptome Sequences
Source: Front Physiol. 2018 May 3;9:383. doi: 10.3389/fphys.2018.00383 (PMC5943558; doi:10.3389/fphys.2018.00383)
Supplement: Supplementary file 3 [file Table_3.DOCX]

**Table 3** Fucational annotation of SPB unigenes

| Annotated Database | Annotated_Number | 300<=length<1000 | length>=1000 |
| --- | --- | --- | --- |
| COG_Annotation | 8503 | 2769 | 4611 |
| GO_Annotation | 15079 | 5426 | 6723 |
| KEGG_Annotation | 8861 | 3363 | 3599 |
| Swissprot_Annotation | 21799 | 7621 | 10282 |
| nr_Annotation | 27628 | 10717 | 11674 |
| All_Annotated | 28338 | 10975 | 11695 |
