# Supplementary material for: Functional Analysis of RNA Interference-Related Soybean Pod Borer (Lepidoptera) Genes Based on Transcriptome Sequences
Source: Front Physiol. 2018 May 3;9:383. doi: 10.3389/fphys.2018.00383 (PMC5943558; doi:10.3389/fphys.2018.00383)
Supplement: Supplementary file 4 [file Table_4.DOCX]

**Table S4** Pathway assignment based on KEGG

| pathway | Pathway ID | Count (8861) | Percentage |
| --- | --- | --- | --- |
| Ribosome | ko03010 | 395 | 4.45773615 |
| RNA transport | ko03013 | 361 | 4.07403228 |
| Protein processing in endoplasmic reticulum | ko04141 | 352 | 3.9724636 |
| Spliceosome | ko03040 | 351 | 3.9611782 |
| Oxidative phosphorylation | ko00190 | 315 | 3.55490351 |
| Purine metabolism | ko00230 | 216 | 2.43764812 |
| Lysosome | ko04142 | 197 | 2.22322537 |
| Endocytosis | ko04144 | 192 | 2.16679833 |
| Ubiquitin mediated proteolysis | ko04120 | 186 | 2.09908588 |
| RNA degradation | ko03018 | 184 | 2.07651507 |
| Phagosome | ko04145 | 177 | 1.99751721 |
| Wnt signaling pathway | ko04310 | 175 | 1.97494639 |
| mRNA surveillance pathway | ko03015 | 157 | 1.77180905 |
| Pyrimidine metabolism | ko00240 | 145 | 1.63638416 |
| Glycolysis / Gluconeogenesis | ko00010 | 126 | 1.4219614 |
| Ribosome biogenesis in eukaryotes | ko03008 | 121 | 1.36553436 |
| Proteasome | ko03050 | 111 | 1.25268028 |
| Citrate cycle (TCA cycle) | ko00020 | 109 | 1.23010947 |
| Glutathione metabolism | ko00480 | 102 | 1.15111161 |
| Peroxisome | ko04146 | 98 | 1.10596998 |
| Arginine and proline metabolism | ko00330 | 96 | 1.08339916 |
| Progesterone-mediated oocyte maturation | ko04914 | 96 | 1.08339916 |
| MAPK signaling pathway | ko04010 | 90 | 1.01568672 |
| Neuroactive ligand-receptor interaction | ko04080 | 89 | 1.00440131 |
| Valine, leucine and isoleucine degradation | ko00280 | 87 | 0.98183049 |
| Pyruvate metabolism | ko00620 | 85 | 0.95925968 |
| Amino sugar and nucleotide sugar metabolism | ko00520 | 84 | 0.94797427 |
| ECM-receptor interaction | ko04512 | 84 | 0.94797427 |
| Oocyte meiosis | ko04114 | 83 | 0.93668886 |
| Regulation of actin cytoskeleton | ko04810 | 83 | 0.93668886 |
| Glycerophospholipid metabolism | ko00564 | 80 | 0.90283264 |
| Cell cycle | ko04110 | 80 | 0.90283264 |
| Lysine degradation | ko00310 | 79 | 0.89154723 |
| Phosphatidylinositol signaling system | ko04070 | 79 | 0.89154723 |
| Tight junction | ko04530 | 73 | 0.82383478 |
| Calcium signaling pathway | ko04020 | 72 | 0.81254937 |
| TGF-beta signaling pathway | ko04350 | 70 | 0.78997856 |
| Inositol phosphate metabolism | ko00562 | 69 | 0.77869315 |
| N-Glycan biosynthesis | ko00510 | 68 | 0.76740774 |
| Alanine, aspartate and glutamate metabolism | ko00250 | 66 | 0.74483693 |
| Cysteine and methionine metabolism | ko00270 | 66 | 0.74483693 |
| Focal adhesion | ko04510 | 66 | 0.74483693 |
| Insulin signaling pathway | ko04910 | 66 | 0.74483693 |
| Fatty acid metabolism | ko00071 | 65 | 0.73355152 |
| Nucleotide excision repair | ko03420 | 64 | 0.72226611 |
| Protein export | ko03060 | 63 | 0.7109807 |
| Starch and sucrose metabolism | ko00500 | 62 | 0.69969529 |
| mTOR signaling pathway | ko04150 | 62 | 0.69969529 |
| Propanoate metabolism | ko00640 | 61 | 0.68840989 |
| RNA polymerase | ko03020 | 61 | 0.68840989 |
| Pentose phosphate pathway | ko00030 | 58 | 0.65455366 |
| Glycerolipid metabolism | ko00561 | 56 | 0.63198285 |
| Drug metabolism - cytochrome P450 | ko00982 | 56 | 0.63198285 |
| Tryptophan metabolism | ko00380 | 55 | 0.62069744 |
| Aminoacyl-tRNA biosynthesis | ko00970 | 55 | 0.62069744 |
| Basal transcription factors | ko03022 | 55 | 0.62069744 |
| Fructose and mannose metabolism | ko00051 | 54 | 0.60941203 |
| Metabolism of xenobiotics by cytochrome P450 | ko00980 | 54 | 0.60941203 |
| DNA replication | ko03030 | 53 | 0.59812662 |
| Galactose metabolism | ko00052 | 52 | 0.58684121 |
| Natural killer cell mediated cytotoxicity | ko04650 | 51 | 0.57555581 |
| Drug metabolism - other enzymes | ko00983 | 49 | 0.55298499 |
| Butanoate metabolism | ko00650 | 48 | 0.54169958 |
| Adherens junction | ko04520 | 48 | 0.54169958 |
| Glycine, serine and threonine metabolism | ko00260 | 47 | 0.53041417 |
| beta-Alanine metabolism | ko00410 | 47 | 0.53041417 |
| Notch signaling pathway | ko04330 | 47 | 0.53041417 |
| Cardiac muscle contraction | ko04260 | 43 | 0.48527254 |
| Biosynthesis of unsaturated fatty acids | ko01040 | 41 | 0.46270173 |
| SNARE interactions in vesicular transport | ko04130 | 41 | 0.46270173 |
| Apoptosis | ko04210 | 40 | 0.45141632 |
| Dorso-ventral axis formation | ko04320 | 40 | 0.45141632 |
| Jak-STAT signaling pathway | ko04630 | 40 | 0.45141632 |
| Porphyrin and chlorophyll metabolism | ko00860 | 38 | 0.4288455 |
| Mismatch repair | ko03430 | 38 | 0.4288455 |
| Sphingolipid metabolism | ko00600 | 37 | 0.41756009 |
| Cell adhesion molecules (CAMs) | ko04514 | 37 | 0.41756009 |
| Homologous recombination | ko03440 | 36 | 0.40627469 |
| Hedgehog signaling pathway | ko04340 | 36 | 0.40627469 |
| Pentose and glucuronate interconversions | ko00040 | 35 | 0.39498928 |
| Other glycan degradation | ko00511 | 34 | 0.38370387 |
| Glyoxylate and dicarboxylate metabolism | ko00630 | 34 | 0.38370387 |
| Base excision repair | ko03410 | 34 | 0.38370387 |
| VEGF signaling pathway | ko04370 | 34 | 0.38370387 |
| GnRH signaling pathway | ko04912 | 34 | 0.38370387 |
| ABC transporters | ko02010 | 33 | 0.37241846 |
| PPAR signaling pathway | ko03320 | 32 | 0.36113305 |
| Melanogenesis | ko04916 | 32 | 0.36113305 |
| Tyrosine metabolism | ko00350 | 31 | 0.34984765 |
| MAPK signaling pathway - fly | ko04013 | 31 | 0.34984765 |
| Fatty acid biosynthesis | ko00061 | 30 | 0.33856224 |
| Nitrogen metabolism | ko00910 | 29 | 0.32727683 |
| ErbB signaling pathway | ko04012 | 29 | 0.32727683 |
| Vascular smooth muscle contraction | ko04270 | 29 | 0.32727683 |
| One carbon pool by folate | ko00670 | 28 | 0.31599142 |
| Glycosaminoglycan degradation | ko00531 | 27 | 0.30470602 |
| Nicotinate and nicotinamide metabolism | ko00760 | 27 | 0.30470602 |
| p53 signaling pathway | ko04115 | 27 | 0.30470602 |
| Toll-like receptor signaling pathway | ko04620 | 27 | 0.30470602 |
| Valine, leucine and isoleucine biosynthesis | ko00290 | 26 | 0.29342061 |
| Retinol metabolism | ko00830 | 26 | 0.29342061 |
| Glycosylphosphatidylinositol(GPI)-anchor biosynthesis | ko00563 | 25 | 0.2821352 |
| Phototransduction - fly | ko04745 | 25 | 0.2821352 |
| Gap junction | ko04540 | 24 | 0.27084979 |
| Other types of O-glycan biosynthesis | ko00514 | 23 | 0.25956438 |
| Terpenoid backbone biosynthesis | ko00900 | 23 | 0.25956438 |
| NOD-like receptor signaling pathway | ko04621 | 23 | 0.25956438 |
| Alzheimer's disease | ko05010 | 23 | 0.25956438 |
| Adipocytokine signaling pathway | ko04920 | 22 | 0.24827898 |
| Fatty acid elongation in mitochondria | ko00062 | 21 | 0.23699357 |
| Ether lipid metabolism | ko00565 | 21 | 0.23699357 |
| Arachidonic acid metabolism | ko00590 | 21 | 0.23699357 |
| Circadian rhythm - mammal | ko04710 | 21 | 0.23699357 |
| Ascorbate and aldarate metabolism | ko00053 | 19 | 0.21442275 |
| Regulation of autophagy | ko04140 | 19 | 0.21442275 |
| Phenylalanine metabolism | ko00360 | 18 | 0.20313734 |
| Glycosphingolipid biosynthesis - globo series | ko00603 | 18 | 0.20313734 |
| Pantothenate and CoA biosynthesis | ko00770 | 18 | 0.20313734 |
| Folate biosynthesis | ko00790 | 18 | 0.20313734 |
| Selenocompound metabolism | ko00450 | 17 | 0.19185194 |
| Linoleic acid metabolism | ko00591 | 17 | 0.19185194 |
| RIG-I-like receptor signaling pathway | ko04622 | 17 | 0.19185194 |
| Huntington's disease | ko05016 | 17 | 0.19185194 |
| Amoebiasis | ko05146 | 17 | 0.19185194 |
| Insect hormone biosynthesis | ko00981 | 16 | 0.18056653 |
| Taurine and hypotaurine metabolism | ko00430 | 15 | 0.16928112 |
| Mucin type O-Glycan biosynthesis | ko00512 | 15 | 0.16928112 |
| Glycosaminoglycan biosynthesis - heparan sulfate | ko00534 | 15 | 0.16928112 |
| Pathways in cancer | ko05200 | 15 | 0.16928112 |
| Ubiquinone and other terpenoid-quinone biosynthesis | ko00130 | 13 | 0.1467103 |
| Histidine metabolism | ko00340 | 13 | 0.1467103 |
| alpha-Linolenic acid metabolism | ko00592 | 13 | 0.1467103 |
| Glycosphingolipid biosynthesis - ganglio series | ko00604 | 13 | 0.1467103 |
| Non-homologous end-joining | ko03450 | 13 | 0.1467103 |
| Renin-angiotensin system | ko04614 | 13 | 0.1467103 |
| Vitamin B6 metabolism | ko00750 | 12 | 0.1354249 |
| Chemokine signaling pathway | ko04062 | 12 | 0.1354249 |
| Cytosolic DNA-sensing pathway | ko04623 | 12 | 0.1354249 |
| Parkinson's disease | ko05012 | 12 | 0.1354249 |
| Bacterial invasion of epithelial cells | ko05100 | 12 | 0.1354249 |
| Glycosphingolipid biosynthesis - lacto and neolacto series | ko00601 | 11 | 0.12413949 |
| Neurotrophin signaling pathway | ko04722 | 11 | 0.12413949 |
| Phenylalanine, tyrosine and tryptophan biosynthesis | ko00400 | 10 | 0.11285408 |
| D-Glutamine and D-glutamate metabolism | ko00471 | 10 | 0.11285408 |
| Cytokine-cytokine receptor interaction | ko04060 | 10 | 0.11285408 |
| Sulfur relay system | ko04122 | 10 | 0.11285408 |
| Amyotrophic lateral sclerosis (ALS) | ko05014 | 10 | 0.11285408 |
| Synthesis and degradation of ketone bodies | ko00072 | 9 | 0.10156867 |
| Steroid biosynthesis | ko00100 | 9 | 0.10156867 |
| Glycosaminoglycan biosynthesis - chondroitin sulfate | ko00532 | 9 | 0.10156867 |
| Leukocyte transendothelial migration | ko04670 | 9 | 0.10156867 |
| Long-term potentiation | ko04720 | 9 | 0.10156867 |
| Glycosaminoglycan biosynthesis - keratan sulfate | ko00533 | 8 | 0.09028326 |
| Sulfur metabolism | ko00920 | 8 | 0.09028326 |
| T cell receptor signaling pathway | ko04660 | 8 | 0.09028326 |
| Vibrio cholerae infection | ko05110 | 8 | 0.09028326 |
| Toxoplasmosis | ko05145 | 8 | 0.09028326 |
| Cyanoamino acid metabolism | ko00460 | 7 | 0.07899786 |
| Phototransduction | ko04744 | 7 | 0.07899786 |
| Salivary secretion | ko04970 | 7 | 0.07899786 |
| Pancreatic secretion | ko04972 | 7 | 0.07899786 |
| Lysine biosynthesis | ko00300 | 6 | 0.06771245 |
| Fc gamma R-mediated phagocytosis | ko04666 | 6 | 0.06771245 |
| Circadian rhythm - fly | ko04711 | 6 | 0.06771245 |
| Protein digestion and absorption | ko04974 | 6 | 0.06771245 |
| Bile secretion | ko04976 | 6 | 0.06771245 |
| Hepatitis C | ko05160 | 6 | 0.06771245 |
| Colorectal cancer | ko05210 | 6 | 0.06771245 |
| Dilated cardiomyopathy | ko05414 | 6 | 0.06771245 |
| Riboflavin metabolism | ko00740 | 5 | 0.05642704 |
| Axon guidance | ko04360 | 5 | 0.05642704 |
| Gastric acid secretion | ko04971 | 5 | 0.05642704 |
| Prostate cancer | ko05215 | 5 | 0.05642704 |
| Caffeine metabolism | ko00232 | 4 | 0.04514163 |
| Complement and coagulation cascades | ko04610 | 4 | 0.04514163 |
| Long-term depression | ko04730 | 4 | 0.04514163 |
| Olfactory transduction | ko04740 | 4 | 0.04514163 |
| Vasopressin-regulated water reabsorption | ko04962 | 4 | 0.04514163 |
| Chagas disease (American trypanosomiasis) | ko05142 | 4 | 0.04514163 |
| Endometrial cancer | ko05213 | 4 | 0.04514163 |
| Systemic lupus erythematosus | ko05322 | 4 | 0.04514163 |
| Hypertrophic cardiomyopathy (HCM) | ko05410 | 4 | 0.04514163 |
| Steroid hormone biosynthesis | ko00140 | 3 | 0.03385622 |
| Butirosin and neomycin biosynthesis | ko00524 | 3 | 0.03385622 |
| Thiamine metabolism | ko00730 | 3 | 0.03385622 |
| Lipoic acid metabolism | ko00785 | 3 | 0.03385622 |
| Fc epsilon RI signaling pathway | ko04664 | 3 | 0.03385622 |
| Taste transduction | ko04742 | 3 | 0.03385622 |
| Prion diseases | ko05020 | 3 | 0.03385622 |
| Basal cell carcinoma | ko05217 | 3 | 0.03385622 |
| Non-small cell lung cancer | ko05223 | 3 | 0.03385622 |
| Arrhythmogenic right ventricular cardiomyopathy (ARVC) | ko05412 | 3 | 0.03385622 |
| Viral myocarditis | ko05416 | 3 | 0.03385622 |
| Osteoclast differentiation | ko04380 | 2 | 0.02257082 |
| Antigen processing and presentation | ko04612 | 2 | 0.02257082 |
| Intestinal immune network for IgA production | ko04672 | 2 | 0.02257082 |
| Type I diabetes mellitus | ko04940 | 2 | 0.02257082 |
| Fat digestion and absorption | ko04975 | 2 | 0.02257082 |
| Renal cell carcinoma | ko05211 | 2 | 0.02257082 |
| Chronic myeloid leukemia | ko05220 | 2 | 0.02257082 |
| Small cell lung cancer | ko05222 | 2 | 0.02257082 |
| Primary bile acid biosynthesis | ko00120 | 1 | 0.01128541 |
| Biotin metabolism | ko00780 | 1 | 0.01128541 |
| B cell receptor signaling pathway | ko04662 | 1 | 0.01128541 |
| Aldosterone-regulated sodium reabsorption | ko04960 | 1 | 0.01128541 |
| Proximal tubule bicarbonate reclamation | ko04964 | 1 | 0.01128541 |
| Vitamin digestion and absorption | ko04977 | 1 | 0.01128541 |
| Leishmaniasis | ko05140 | 1 | 0.01128541 |
| African trypanosomiasis | ko05143 | 1 | 0.01128541 |
| Malaria | ko05144 | 1 | 0.01128541 |
| Pancreatic cancer | ko05212 | 1 | 0.01128541 |
| Glioma | ko05214 | 1 | 0.01128541 |
| Thyroid cancer | ko05216 | 1 | 0.01128541 |
| Autoimmune thyroid disease | ko05320 | 1 | 0.01128541 |
| Allograft rejection | ko05330 | 1 | 0.01128541 |
| Graft-versus-host disease | ko05332 | 1 | 0.01128541 |
