# Supplementary material for: Functional Analysis of RNA Interference-Related Soybean Pod Borer (Lepidoptera) Genes Based on Transcriptome Sequences
Source: Front Physiol. 2018 May 3;9:383. doi: 10.3389/fphys.2018.00383 (PMC5943558; doi:10.3389/fphys.2018.00383)
Supplement: Supplementary file 5 [file Table_5.DOCX]

**Table S5** Overview of identified genes related to the RNAi pathways in SBP

| Group | RNAi-related gene | Contig | gene expression |
| --- | --- | --- | --- |
| RNAi core machinery: siRNA pathway | Dicer2 | c79492.graph_c0 | up* |
|  | R2D2 | c87907.graph_c0 | - |
|  | Ago2 | c65749.graph_c0 | up* |
| RNAi core machinery: miRNA pathway | DGCR8 | c79402.graph_c0 | - |
|  | Dicer1 | c79871.graph_c0 | - |
|  | Loquacious | c71406.graph_c0 | - |
|  | Ago1 | c70013.graph_c0 | - |
|  | Piwi/Aubergine | c63841.graph_c0 | - |
| RNAi core machinery: piRNA pathway | Ago3 | c79403.graph_c0 | - |
|  | Translin | c77477.graph_c3 | - |
| Auxiliary factors (RISC) | Gawky | c74233.graph_c0 | - |
|  | FXMR | c71444.graph_c0 | - |
|  | Belle | c34167.graph_c0 | - |
|  | p68 RNA helicase | c34167.graph_c0 | up* |
|  | Armitage | c76626.graph_c0 | - |
|  | Staufen | c76294.graph_c0 | - |
|  | Maelstrom | c76626.graph_c0 | - |
|  | PRMT5 | c78921.graph_c0 | - |
|  | HPS4 | c74864.graph_c0 | - |
| DsRNA uptake | FBX011 | c68483.graph_c0 | - |
|  | vacuolar H-ATPase 16 | c84790.graph_c0 | - |
|  | Sil1 | c79601.graph_c0 | up |
|  | Sil2 | c79695.graph_c0 | - |
|  | Sil3 | c77135.graph_c0 | - |
|  | Src | c76935.graph_c0 | up |
|  | Srb1 | c61934..graph_c0 | - |
|  | Srb2 | c66328.graph_c0 | - |
|  | Srb3 | c74700.graph_c0 | - |
|  | Srb4 | c75587.graph_c0 | up |
|  | Srb5 | c76572.graph_c0 | - |
|  | Srb6 | c76936.graph_c0 | - |
|  | Ars2-RA | c78781.graph_c0 | - |
| Antiviral RNAi | Ars2-RB | c76696.graph_c0 | - |
|  | Egalitarian | c71666.graph_c0 | - |
| Nuclease | Oligoribonuclease | c76361.graph_c0 | - |
|  | Snipper | c81830.graph_c0 | - |

*corresponds to Relative expression of gene significantly higher in larvae feeding on transgenic dsSpbP0 soybean Bean pods than ‘DN50’ soybean Bean pods.
