# Supplementary material for: Functional Analysis of RNA Interference-Related Soybean Pod Borer (Lepidoptera) Genes Based on Transcriptome Sequences
Source: Front Physiol. 2018 May 3;9:383. doi: 10.3389/fphys.2018.00383 (PMC5943558; doi:10.3389/fphys.2018.00383)
Supplement: Supplementary file 6 [file Table_6.DOCX]

**Table S6** Primer used in dsRNA synthesis and qPCR amplification.

| Names | Sequences |
| --- | --- |
| Yy-750T7-F | 5’-GGATCCTAATACGACTCACTATAGGGAGAATGGCCTCAACACTGTC-3' |
| Yy-750T7-R | 5’ -GGATCCTAATACGACTCACTATAGGGCGTTCTGCATCAATTTTCA-3' |
| La2-750T7-F | 5’ -GGATCCTAATACGACTCACTATAGGGTTGGCTGATGGAGTCGAAC-3' |
| La2-750T7-R | 5’-GGATCCTAATACGACTCACTATAGGTACGCACCAGGGATGTTGT-3' |
| Eb-750T7F | 5’-GGATCCTAATACGACTCACTATAGGCTCATGCAACTAGCGCAGAC-3' |
| Eb-750T7 R | 5’-GGATCCTAATACGACTCACTATAGGCGTCTTTATGCTCATCTCGC-3' |
| GFPT7F | 5’-GGATCCTAATACGACTCACTATAGGGGTGATGCTACATACGGAAAG-3' |
| GFPT7 R | 5’-GGATCCTAATACGACTCACTATAGGTTGTTTGTCTGCCGTGAT-3' |
| La2-500T7F | 5’-GGATCCTAATACGACTCACTATAGGATATTTAGTTGCCCCGCAAG-3' |
| La2-500T7 R | 5’-GGATCCTAATACGACTCACTATAGGCGATGTGGAAGAGGAAGTGG-3' |
| La2-300T7F | 5’-GGATCCTAATACGACTCACTATAGGCTAGAGCGTGCCGAGAAAAT-3' |
| La2-300T7 R | 5’-GGATCCTAATACGACTCACTATAGGATCCTCTTTGCCAGATTCCA-3' |
| Dcr2T7F | 5’-GGATCCTAATACGACTCACTATAGGATTCTCCTGGAGCGGCTTA-3' |
| Dcr 2T7 R | 5’-GGATCCTAATACGACTCACTATAGGCAGGATTTTGGCGGTGAAT-3' |
| Ago2T7F | 5’-GGATCCTAATACGACTCACTATAGGGCAAGGAGATAGACCCGCTT-3' |
| Ago2T7 R | 5’-GGATCCTAATACGACTCACTATAGGTCATTGTCGGATACATTCGG-3' |
| Sil1T7 F | 5’-GGATCCTAATACGACTCACTATAGGTCGTTTATCGTGAGCGTTGT-3' |
| Sil1T7 R | 5’-GGATCCTAATACGACTCACTATAGGAGACTGGGTGTTCACCGTCA-3' |
| Sil2T7 F | 5’-GGATCCTAATACGACTCACTATAGGCGAGCCAGCCGAAATACTAC-3' |
| Sil2T7 R | 5’-GGATCCTAATACGACTCACTATAGGTTGTCGAATACTGGGCACG-3' |
| Sil3T7 F | 5’-GGATCCTAATACGACTCACTATAGGATCACACAGACGGCACCTC-3' |
| Sil3T7 R | 5’-GGATCCTAATACGACTCACTATAGGCGCGGACCACTGCTATTACT-3' |
| Src T7 R | 5’-GGATCCTAATACGACTCACTATAGGCCACGTCTCTCCGTACCTCT-3' |
| Src T7 R | 5’-GGATCCTAATACGACTCACTATAGGCCACGTCTCTCCGTACCTCT-3' |
| Srb1T7 F | 5’-GGATCCTAATACGACTCACTATAGGTTCTGACTGTGGTTTTGGGG-3' |
| Srb1T7 R | 5’-GGATCCTAATACGACTCACTATAGGTGCTTCTGGATACGCTTCCT-3' |
| Srb2T7 F | 5’-GGATCCTAATACGACTCACTATAGGGGACTGGGGCTTCTTGGAG-3' |
| Srb2T7 R | 5’-GGATCCTAATACGACTCACTATAGGAGGCTGTCCTCGTAGTTCCA-3' |
| Srb3T7 F | 5-GGATCCTAATACGACTCACTATAGGGCAACTGCAGAAGATGATGG-3' |
| Srb3T7 R | 5-GGATCCTAATACGACTCACTATAGGCCACGTCCGCTGGTTATAGT-3' |
| Srb4T7 F | 5-GGATCCTAATACGACTCACTATAGGGCTTACCCGGACGACCTAAC-3' |
| Srb4T7 R | 5-GGATCCTAATACGACTCACTATAGGGCCTTCGATACGTTCGTACA-3' |
| Srb5T7 F | 5-GGATCCTAATACGACTCACTATAGGAAAACGTGACGGGATTGAAC-3' |
| Srb5T7 R | 5-GGATCCTAATACGACTCACTATAGGGTTGAGAATGTGCCGTGGTA-3' |
| Srb6T7 F | 5-GGATCCTAATACGACTCACTATAGGCAACCACGACCGGTACATTA-3' |
| Srb6T7 R | 5-GGATCCTAATACGACTCACTATAGGTTTAGGAAGAAGTCGCTCTGG-3' |
| Lac2-F | 5-GTGAGTACCAGCGTTTCCTTG-3' |
| Lac2-R | 5-TGATGGAGTCGAACGTGGTA-3' |
| Dcr 2-F | 5-TAGACCGAGTGACAATGCTG-3' |
| Dcr 2-R | 5-TAACGCCTGAAGCGGTCCCT-3' |
| Ago2-F | 5-ATGGCTAAAGGGAAAAAGAAG-3' |
| Ago2-R | 5-CTAAACAAAGAACATGCGAC-3' |
| Sil1-F | 5-CGTTACCGCCACACAGCAGAAAG-3' |
| Sil1-R | 5-TCGTCCGCCATCGCCAGT-3' |
| Sil2-F | 5-ACACCATAATTTCACTCCGC-3' |
| Sil2-R | 5-TGGGTGTTCATCGTGCTAAC-3' |
| Sil3-F | 5-CGCGGACCACTGCTATTACT-3' |
| Sil3-R | 5-ATCACACAGACGGCACCTC-3' |
| Src-F | 5-TTTAGAGCAAAACCAAAGATGTCCG-3' |
| Src-R | 5-TTCAGTCTTTACTCGATGATCAATT-3' |
| Srb1-F | 5-CAGGTGGAACTGGGAAGG-3' |
| Srb1-R | 5-CACCCAGACATCCTCTAACA-3' |
| Srb2-F | 5-AGCACTGAACCCAAATAC-3' |
| Srb2-R | 5-TGCCTTGCAGCTTACAC-3' |
| Srb3-F | 5-AAATCAGGAATCACAACAAGGAAGC-3' |
| Srb3-R | 5-ATGTATTAAGTATTGATAACTGGAA-3' |
| Srb4-F | 5-GAGATGGCGTTCAACACTAC-3' |
| Srb4-R | 5-ATGCTCCTCACAAGAAACTA-3' |
| Srb5-F | 5-TAAAAAAACTTGTGTAACTAAA-3' |
| Srb5-R | 5-AAACTCCCATTAGTCAGAAC-3' |
| Srb6-F | 5-CGCTCTATCCACGTCTCTCC-3' |
| Srb6-R | 5-CTGTTTACTGGGCCAGCGTA-3' |
| actin-F | 5-GTTATGATGCCCTACACCG-3' |
| actin-R | 5-CACTTCGCTGAGTTTACG-3' |

Underlined sequence corresponds to T7 promoter; F, forward; R, reverse.
