# Supplementary material for: Functional Analysis of RNA Interference-Related Soybean Pod Borer (Lepidoptera) Genes Based on Transcriptome Sequences
Source: Front Physiol. 2018 May 3;9:383. doi: 10.3389/fphys.2018.00383 (PMC5943558; doi:10.3389/fphys.2018.00383)
Supplement: Supplementary file 7 [file Presentation_1.PDF]

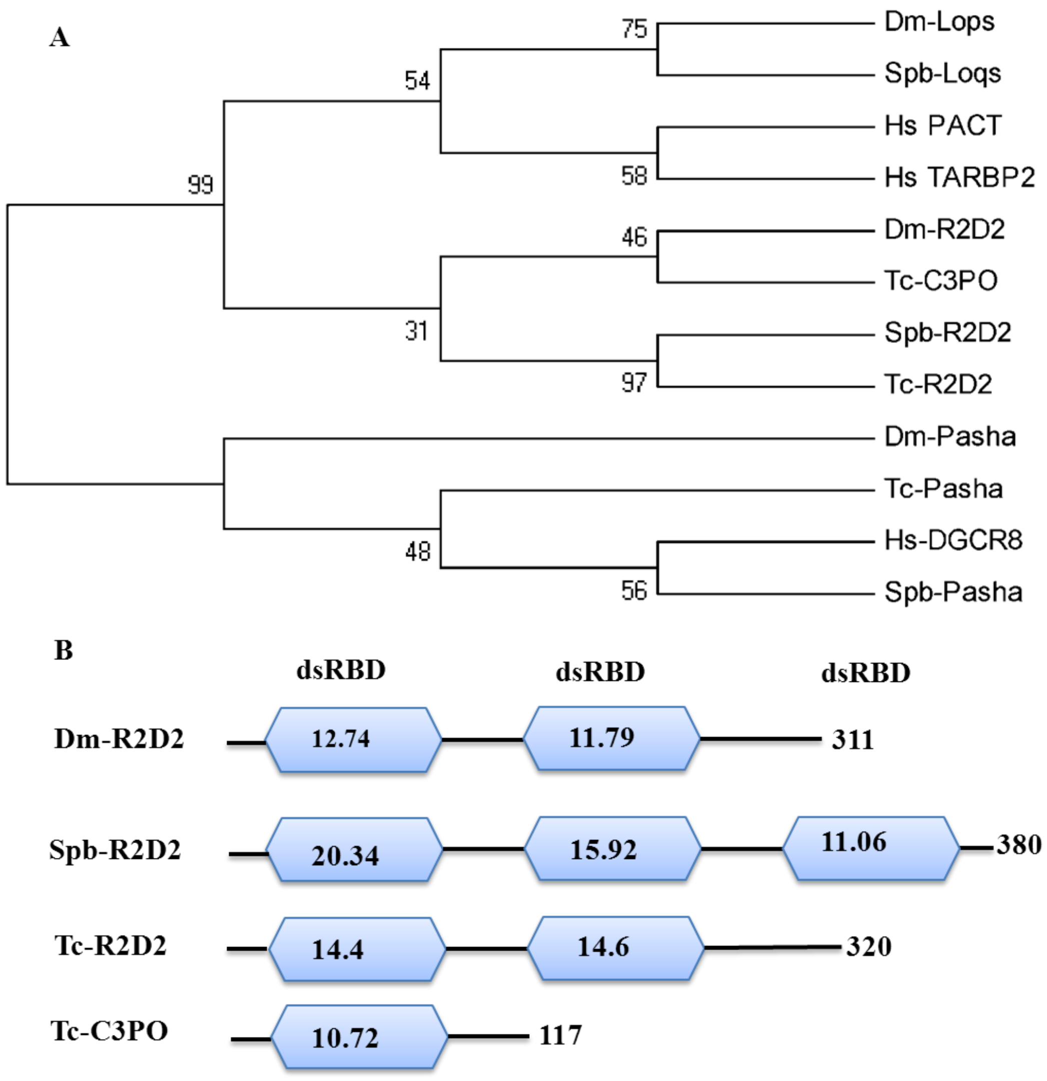

Figure S1 Phylogenetic and domain architecture analysis of dsRNA binding proteins (A) Neighbor-joining inferred phylogeny of insect Dicer proteins, including DmLops (NP_723813), SpbLops (c71406), HsPACT (NP_003681), HsTARBP2 (NP_599150), DmR2D2 (NP_ 609152), TcR2D2 (NP_001128425), TcC3PO (ABX72055), SpbR2D2 (c19500), DmPasha (NP_723813), DmPasha (NP_651879), TcPasha (XP_971282), HsDGCR8 (AF165527), SpbR2D2 (c79402). Bootstrap values (1000 replicates) are shown next to the branches. (B) Domain architecture of dsRNA binding proteins proteins (R2D2), dsRNA-binding domain are indicated. Bm, *Bombyx mori*; Dm, *Drosophila melanogaster*; Tc, Tribolium castaneum; Hs, *Homo sapiens*;

Spb, soybean pod borer.
