# Supplementary material for: Functional Analysis of RNA Interference-Related Soybean Pod Borer (Lepidoptera) Genes Based on Transcriptome Sequences
Source: Front Physiol. 2018 May 3;9:383. doi: 10.3389/fphys.2018.00383 (PMC5943558; doi:10.3389/fphys.2018.00383)
Supplement: Supplementary file 8 [file Presentation_2.PDF]

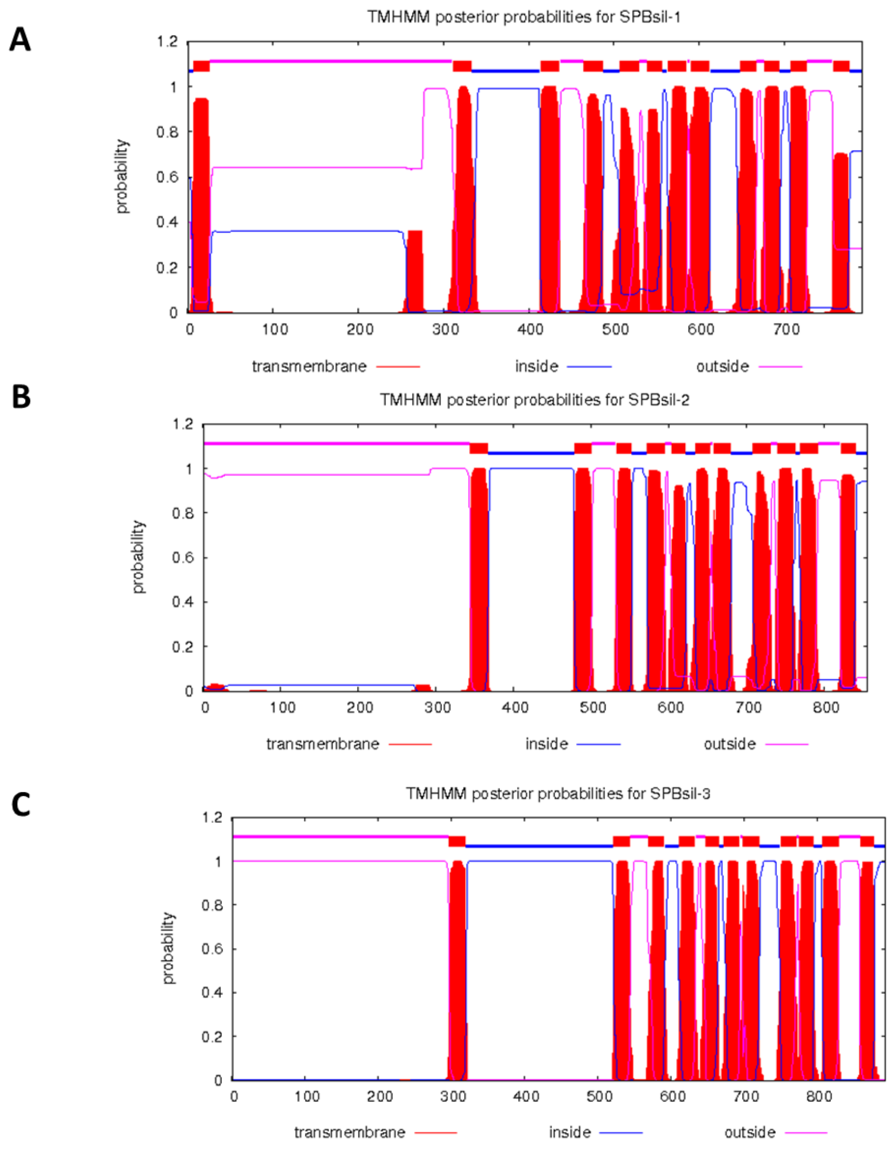

Figure S2 SbpSil Proteins were analyzed by TMHMMserver 2.0. (A) SbpSil1 Protein (C77135) was analyzed by TMHMMserver 2.0 (B) SbpSil2 Protein (C79601) was analyzed by TMHMMserver 2.0 (C) SbpSil3 Protein (C79695) was analyzed by TMHMMserver 2.0.
